# Discovery of drug–omics associations in type 2 diabetes with generative deep-learning models

**DOI:** 10.1038/s41587-022-01520-x

**Published:** 2023-01-02

**Authors:** Rosa Lundbye Allesøe, Agnete Troen Lundgaard, Ricardo Hernández Medina, Alejandro Aguayo-Orozco, Joachim Johansen, Jakob Nybo Nissen, Caroline Brorsson, Gianluca Mazzoni, Lili Niu, Jorge Hernansanz Biel, Cristina Leal Rodríguez, Valentas Brasas, Henry Webel, Michael Eriksen Benros, Anders Gorm Pedersen, Piotr Jaroslaw Chmura, Ulrik Plesner Jacobsen, Andrea Mari, Robert Koivula, Anubha Mahajan, Ana Vinuela, Juan Fernandez Tajes, Sapna Sharma, Mark Haid, Mun-Gwan Hong, Petra B. Musholt, Federico De Masi, Josef Vogt, Helle Krogh Pedersen, Valborg Gudmundsdottir, Angus Jones, Gwen Kennedy, Jimmy Bell, E. Louise Thomas, Gary Frost, Henrik Thomsen, Elizaveta Hansen, Tue Haldor Hansen, Henrik Vestergaard, Mirthe Muilwijk, Marieke T. Blom, Leen M. ‘t Hart, Francois Pattou, Violeta Raverdy, Soren Brage, Tarja Kokkola, Alison Heggie, Donna McEvoy, Miranda Mourby, Jane Kaye, Andrew Hattersley, Timothy McDonald, Martin Ridderstråle, Mark Walker, Ian Forgie, Giuseppe N. Giordano, Imre Pavo, Hartmut Ruetten, Oluf Pedersen, Torben Hansen, Emmanouil Dermitzakis, Paul W. Franks, Jochen M. Schwenk, Jerzy Adamski, Mark I. McCarthy, Ewan Pearson, Karina Banasik, Simon Rasmussen, Søren Brunak, IMI DIRECT Consortium, Philippe Froguel, Cecilia Engel Thomas, Ragna Haussler, Joline Beulens, Femke Rutters, Giel Nijpels, Sabine van Oort, Lenka Groeneveld, Petra Elders, Toni Giorgino, Marianne Rodriquez, Rachel Nice, Mandy Perry, Susanna Bianzano, Ulrike Graefe-Mody, Anita Hennige, Rolf Grempler, Patrick Baum, Hans-Henrik Stærfeldt, Nisha Shah, Harriet Teare, Beate Ehrhardt, Joachim Tillner, Christiane Dings, Thorsten Lehr, Nina Scherer, Iryna Sihinevich, Louise Cabrelli, Heather Loftus, Roberto Bizzotto, Andrea Tura, Koen Dekkers, Nienke van Leeuwen, Leif Groop, Roderick Slieker, Anna Ramisch, Christopher Jennison, Ian McVittie, Francesca Frau, Birgit Steckel-Hamann, Kofi Adragni, Melissa Thomas, Naeimeh Atabaki Pasdar, Hugo Fitipaldi, Azra Kurbasic, Pascal Mutie, Hugo Pomares-Millan, Amelie Bonnefond, Mickael Canouil, Robert Caiazzo, Helene Verkindt, Reinhard Holl, Teemu Kuulasmaa, Harshal Deshmukh, Henna Cederberg, Markku Laakso, Jagadish Vangipurapu, Matilda Dale, Barbara Thorand, Claudia Nicolay, Andreas Fritsche, Anita Hill, Michelle Hudson, Claire Thorne, Kristine Allin, Manimozhiyan Arumugam, Anna Jonsson, Line Engelbrechtsen, Annemette Forman, Avirup Dutta, Nadja Sondertoft, Yong Fan, Stephen Gough, Neil Robertson, Nicky McRobert, Agata Wesolowska-Andersen, Andrew Brown, David Davtian, Adem Dawed, Louise Donnelly, Colin Palmer, Margaret White, Jorge Ferrer, Brandon Whitcher, Anna Artati, Cornelia Prehn, Jonathan Adam, Harald Grallert, Ramneek Gupta, Peter Wad Sackett, Birgitte Nilsson, Konstantinos Tsirigos, Rebeca Eriksen, Bernd Jablonka, Mathias Uhlen, Johann Gassenhuber, Tania Baltauss, Nathalie de Preville, Maria Klintenberg, Moustafa Abdalla

**Affiliations:** 1 Novo Nordisk Foundation Center for Protein Research, Faculty of Health and Medical Sciences, University of Copenhagen, Copenhagen, Denmark; 2 Department of Health Technology, Technical University of Denmark, Kongens Lyngby, Denmark; 3 Copenhagen Research Centre for Mental Health, Mental Health Centre Copenhagen, Copenhagen University Hospital, Copenhagen, Denmark; 4 Department of Immunology and Microbiology, Faculty of Health and Medical Sciences, University of Copenhagen, Copenhagen, Denmark; 5 C.N.R. Institute of Neuroscience, Padova, Italy; 6 Wellcome Centre for Human Genetics, University of Oxford, Oxford, UK; 7 Department of Genetic Medicine and Development, University of Geneva Medical School, Geneva, Switzerland; 8 Biosciences Institute, Faculty of Medical Sciences, Newcastle University, Newcastle, UK; 9 Research Unit of Molecular Epidemiology, Helmholtz Zentrum München, German Research Center for Environmental Health, Neuherberg, Bavaria Germany; 10 Institute of Epidemiology, Helmholtz Zentrum München, German Research Center for Environmental Health, Neuherberg, Bavaria Germany; 11 Chair of Food Chemistry and Molecular and Sensory Science, Technical University of Munich, Freising, Germany; 12 Metabolomics and Proteomics Core, Helmholtz Zentrum Muenchen, German Research Center for Environmental Health, Neuherberg, Germany; 13 Affinity Proteomics, Science for Life Laboratory, School of Engineering Sciences in Chemistry, Biotechnology and Health, KTH Royal Institute of Technology, Solna, Sweden; 14 Research and Development Global Development, Translational Medicine and Clinical Pharmacology, Sanofi-Aventis Deutschland, Frankfurt, Germany; 15 Novo Nordisk Foundation Center for Basic Metabolic Research, Faculty of Health and Medical Sciences, University of Copenhagen, Copenhagen, Denmark; 16 University of Exeter Medical School, Exeter, UK; 17 The Immunoassay Biomarker Core Laboratory, School of Medicine, University of Dundee, Dundee, UK; 18 Research Centre for Optimal Health, Department of Life Sciences, University of Westminster, London, UK; 19 Section for Nutrition Research, Faculty of Medicine, Imperial College London, London, UK; 20 Department of Radiology, Copenhagen University Hospital Herlev-Gentofte, Herlev, Denmark; 21 Department of Epidemiology and Data Science, Amsterdam Public Health Research Institute, Amsterdam UMC, Vrije Universiteit Amsterdam, Amsterdam, the Netherlands; 22 Department of General Practice, Amsterdam Public Health Research Institute, Amsterdam UMC, Vrije Universiteit Amsterdam, Amsterdam, the Netherlands; 23 Department of Biomedical Data Science, Section Molecular Epidemiology, Leiden University Medical Center, Leiden, the Netherlands; 24 Department of Cell and Chemical Biology, Leiden University Medical Center, Leiden, the Netherlands; 25 Inserm, Univ Lille, CHU Lille, Lille Pasteur Institute, EGID, Lille, France; 26 MRC Epidemiology Unit, University of Cambridge School of Clinical Medicine, Cambridge, UK; 27 Department of Medicine, University of Eastern Finland, Kuopio, Finland; 28 Institute of Cellular Medicine, Newcastle University, Newcastle, UK; 29 Diabetes Research Network, Royal Victoria Infirmary, Newcastle, UK; 30 Centre for Health, Law and Emerging Technologies (HeLEX), Faculty of Law, University of Oxford, Oxford, UK; 31 Lund University Diabetes Centre, Department of Clinical Sciences, Lund University, Malmö, Sweden; 32 Translational and Clinical Research Institute, Faculty of Medical Sciences, Newcastle University, Newcastle, UK; 33 Division of Population Health & Genomics, School of Medicine, University of Dundee, Dundee, UK; 34 Genetic and Molecular Epidemiology Unit, Lund University Diabetes Centre, Department of Clinical Sciences, CRC, Lund University, SUS, Malmö, Sweden; 35 Eli Lilly Regional Operations, Vienna, Austria; 36 Harvard T.H. Chan School of Public Health, Boston, MA USA; 37 OCDEM, Radcliffe Department of Medicine, University of Oxford, Oxford, UK; 38 Institute of Experimental Genetics, Helmholtz Zentrum München, German Research Center for Environmental Health, Neuherberg, Germany; 39 Department of Biochemistry, Yong Loo Lin School of Medicine, National University of Singapore, Singapore, Singapore; 40 Institute of Biochemistry, Faculty of Medicine, University of Ljubljana, Ljubljana, Slovenia; 41 Oxford Centre for Diabetes, Endocrinology and Metabolism, University of Oxford, Oxford, UK; 42 Present Address: Genentech, South San Francisco, CA USA; 43 Department of Metabolism, Digestion and Reproduction, Imperial College London, London, UK; 44 Biophysics Institute (IBF-CNR), National Research Council of Italy, Milan, Italy; 45 Department of Biosciences, University of Milan, Milan, Italy; 46 Biotech & Biomarkers Research Department, Institut de Recherches Internationales Servier, Croissy sur Seine, France; 47 Blood Sciences, Royal Devon and Exeter NHS Foundation Trust, Exeter, UK; 48 Boehringer Ingelheim International, Therapeutic Area CardioMetabolism and Respiratory Medicine, Ingelheim am Rhein, Germany; 49 Boehringer Ingelheim International, Therapeutic Area CNS, Retinopathies and Emerging Areas, Ingelheim am Rhein, Germany; 50 Boehringer Ingelheim International, Medicine Cardiometabolism and Respiratory, Biberach an der Riss, Germany; 51 Boehringer Ingelheim International, Translational Medicine & Clinical Pharmacology, Biberach an der Riss, Germany; 52 Centre for Mathematics and Algorithms for Data, University of Bath, Bath, UK; 53 Clinical Operations, Sanofi-Aventis Deutschland, Frankfurt, Germany; 54 Clinical Pharmacy, Saarland University, Saarbrücken, Germany; 55 Clinical Research Centre, Ninewells Hospital and Medical School, University of Dundee, Dundee, Scotland UK; 56 Department of Mathematical Sciences, University of Bath, Bath, UK; 57 Digital and Data Sciences, Sanofi-Aventis Deutschland, Frankfurt, Germany; 58 Eli Lilly and Company, Indianapolis, IN USA; 59 Institute for Epidemiology and Medical Biometry, ZIBMT, University of Ulm, Ulm, Germany; 60 Institute of Biomedicine, Bioinformatics Center, University of Eastern Finland, Kuopio, Finland; 61 Institute of Clinical Medicine, Internal Medicine, University of Eastern Finland, Kuopio, Finland; 62 German Center for Diabetes Research, München-Neuherberg, Germany; 63 Lilly Deutschland, Bad Homburg, Germany; 64 Medizinische Universitätsklinik Tübingen, Eberhard Karls Universität Tübingen, Tübingen, Germany; 65 NIHR Exeter Clinical Research Facility, University of Exeter Medical School, Exeter, UK; 66 Regulatory Genomics and Diabetes, Centre for Genomic Regulation, CIBERDEM, Barcelona, Spain; 67 Strategy and Innovation, Sanofi-Aventis Deutschland, Frankfurt, Germany; 68 Systems Biology, Science for Life Laboratory, School of Engineering Sciences in Chemistry, Biotechnology and Health, KTH Royal Institute of Technology, Solna, Sweden; 69 TMED, Sanofi-Aventis Deutschland, Frankfurt, Germany; 70 Translational and Clinical Research, Metabolism Innovation Pole, Institut de Recherches Internationales Servier, Suresnes Cedex, France

**Keywords:** Data integration, Systems biology, Type 2 diabetes, Machine learning

## Abstract

The application of multiple omics technologies in biomedical cohorts has the potential to reveal patient-level disease characteristics and individualized response to treatment. However, the scale and heterogeneous nature of multi-modal data makes integration and inference a non-trivial task. We developed a deep-learning-based framework, multi-omics variational autoencoders (MOVE), to integrate such data and applied it to a cohort of 789 people with newly diagnosed type 2 diabetes with deep multi-omics phenotyping from the DIRECT consortium. Using in silico perturbations, we identified drug–omics associations across the multi-modal datasets for the 20 most prevalent drugs given to people with type 2 diabetes with substantially higher sensitivity than univariate statistical tests. From these, we among others, identified novel associations between metformin and the gut microbiota as well as opposite molecular responses for the two statins, simvastatin and atorvastatin. We used the associations to quantify drug–drug similarities, assess the degree of polypharmacy and conclude that drug effects are distributed across the multi-omics modalities.

## Abstract (web summary)

Clinical multi-omics data are integrated and analyzed using a generative deep-learning model.

## Main

Drug-response patterns in individuals with complex disease, such as type 2 diabetes (T2D), are intricate. Multiple organs and confounders are typically involved including comorbidities and polypharmacy^1,2^. Conversely, treatment with one or more drugs and the associated polypharmacy effects can have considerable impact on the molecular profile of the individual; however, such changes are still largely unknown^3^. The increasing availability of deep phenotyping and multi-omics screening has proven to be beneficial in the characterization of T2D and other diseases^4–7^, and offer the opportunity to gain mechanistic insights on the action of drugs on disease processes.

Cohort studies can be highly useful for investigating associations between drugs and molecular phenotypes, and can be used to tailor the design of randomized control studies to assess direct causal relationships^8^. Common approaches to analysis of cohort data apply univariate statistical methods, linear and logistic regression, dimensionality reduction and clustering analyses. However, when expanding to multi-omics data such analyses are not straightforward and traditional methods of data interpretation are insufficient to exploit the full scope of multi-modality data.

Here we investigate vertical data integration, where multiple omics datasets have been generated for the same samples. Challenges that must be overcome include integration of data across multiple continuous and discrete data modalities, efficient handling of missing data or even large missing parts of specific data types, differences in dimensionality, modality-specific noise and how to extract associations across data modalities^9–11^. There are several strategies for vertical integration of multi-modal datasets, such as element-wise addition of one dataset at a time, learning individual representations for each dataset before fusion, or multi-dimensional fusion where representations are learned from the input data altogether^9,12–14^. Examples are multi-omics factor analysis (MOFA), iCluster, and data integration analysis for biomarker discovery using latent components (DIABLO) implemented in mixOmics, which can integrate multiple modalities^11,14–16^. However, these methods primarily focus on discovering factors or latent variables that can be used for visualization, clustering, or prediction of disease.

We have previously developed a deep-learning framework on the basis of variational autoencoders (VAE)^17,18^ for integration and binning of large amounts of unstructured metagenomics data^19^. Specifically, a VAE is based on deep neural networks and learns to transform high-dimensional data into a lower-dimensional space, termed a latent representation. During this process the two networks of the VAE learn the structure of input data and associations between the input variables. In our previous study, we found that the VAE could learn to integrate two datasets without any prior knowledge or statistical model^19^. Similarly, others have shown the capabilities of VAEs as integrative models for extracting the underlying signal in data for improving clustering and prediction^12,20–23^, as well as for handling large proportions of missing data^24^. We, therefore, speculated that such a model could be used to integrate even deeper cohort-level multi-omics datasets. While previous studies have primarily focused on stratifying patients using the underlying latent representation^22,25,26^ we were also interested in whether we could acquire insights into the complex relationships that the network learns through data integration.

For this purpose, we exploited that the decoder of the VAE is a generative model. Thus, the final trained decoder will be able to generate new examples of data from the learned latent distribution. On the basis of this principle, a variety of generative models have been used to generate new examples of data, such as single-cell RNA data and artificial human chromosomes^27,28^. Additionally, when combined with Bayesian decision theory they have been used for analysis of single-cell RNA data on the basis of variational inference^29–31^. Generative models also allow investigation of the effect that a virtual perturbation of the input data will have on the generated examples. For instance, Yeo et al. trained a generative model on single-cell RNA time-series data and then perturbed the input data to identify the effect of the perturbation on the output of the generative model^32^. Similarly, a recent study used the generative model of a VAE trained on protein evolutionary data to predict the effect that genetic variants have on the fitness of human proteins^33^. For our multi-modal data, we hypothesized that the generative ability of the VAE would allow us to identify associations between, for example, patient exposures and omics features.

We therefore developed a framework that is based on VAEs that we applied to a cohort of 789 people with newly diagnosed T2D with extensive multi-omics characterization. These modalities included genomics, transcriptomics, proteomics, metabolomics, and microbiomes as well as data on medication, diet questionnaires, and clinical measurements. Our method was able to integrate multi-omics data with clinical and categorical data and was resistant to systematic biases in the data as well as large amounts of missing data. Using an ensemble of generative VAE models, feature perturbation, univariate statistical methods, and Bayesian decision theory we identify cross omics associations. We compared the drug multi-omics profiles and showed that different drugs are associated with unique clinical and molecular profiles. Our method, multi-omics variational autoencoders (MOVE) is freely available, easily scalable, can integrate any number of categorical and continuous datasets, and able to identify features to multi-omics associations.

## Results

### Designing a VAE for multi-omics data integration

**Fig. 1:**
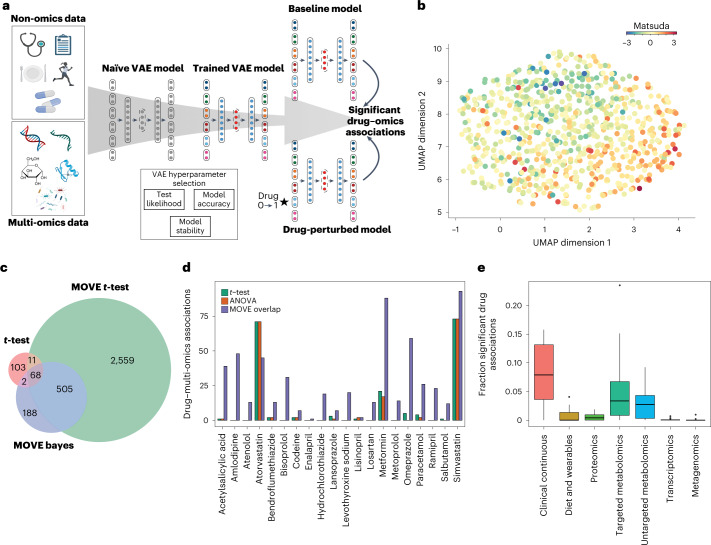
Integrating multi-omics data with a VAE. **a**, Principle of integration and analysis approach using MOVE. Individual-level non-omics and multi-omics data were used as input to a VAE. The optimal network hyperparameters were estimated from the summed test set error across all individuals in the test (test likelihood), training reconstruction accuracy, and model stability. Significant drug–omics associations were identified by perturbing drug status from no (0) to yes (1) for all individuals that were not already administered the drug. **b**, UMAP representation of the latent representation from the 789 people with newly diagnosed T2D. Individuals were colored according to their *z*-scaled Matsuda index from low (blue), average (yellow), and high (red). **c**, Overlap in significant drug–omics associations between standard *t*-test (two-sided, Benjamini–Hochberg FDR < 0.01) on the input data, MOVE *t*-test (multi-stage Bonferroni-corrected, *P* adjust < 0.05) and MOVE Bayes approaches (FDR Bayes < 0.05). The different methods of multiple testing correction corresponded to FDR of 0.05 on the ground-truth dataset. The overlap between MOVE *t*-test and MOVE Bayes was used for further analysis (*n* = 573). **d**, The number of significant associations found between drugs and features in the multi-omics datasets using MOVE *t*-test and MOVE Bayes (purple), *t*-test (green) or ANOVA (orange). See **c** for information on the tests. **e**, Fraction of features in the multi-omics datasets that was found by MOVE to be significantly associated with at least one drug (*n* = 20). The lower and upper hinges correspond to the first and third quartiles. The upper and lower whiskers extend from the hinge to the highest and lowest values, respectively, but no further than 1.5× interquartile range from the hinge. Data beyond the ends of whiskers are outliers and are plotted individually. Source data

We used a dataset of 789 newly diagnosed T2D individuals with extensive multi-omics characterization (Supplementary Table 1). In total the data included 8,807 variables per individual with median missingness within an omics dataset of less than 5% except for metagenomics data where two thirds of the individuals (532) did not have any data (Supplementary Data 1 and Supplementary Fig. 1). Therefore, these individuals had up to 24.7% missingness across the multi-omics data. For the clinical data missingness was higher with a per individual median of 14% and 7% for continuous and categorical clinical data, respectively. We designed the MOVE framework to be flexible in relation to the number of input data types and to be able to handle both continuous and categorical features (Fig. 1a). To identify the optimal hyperparameters that would capture the structure of the data without losing the ability to generalize on unseen individuals, we initially divided the dataset into training and test sets. We then measured the ability of the models to reconstruct the input as well as the stability when refitting the model to the data several times (Supplementary Figs. 2–4). The median reconstruction accuracies were between 0.95–1 and the final models were highly stable when retrained five times with average change of cosine similarities in the latent space of 0.037. Thus, the VAE models were able to reconstruct the data with high accuracy across the individuals (Supplementary Fig. 5).

### The latent space contains important clinical signatures

To illustrate how well the model captured the structure of the clinical data, we analyzed the neural network weights connected to the input variables of the encoder. Here we found the majority of the clinical and dietary variables to be among the top 50 most important (Supplementary Fig. 6). This was also the case when we investigated how the continuous features impacted the positioning of the individuals in the latent space using a Shapley additive explanation (SHAP) analysis^34^, whereas for discrete features we found T2D-associated genetic variants as well as clinically related features to be important (Supplementary Fig. 7). Then, we investigated how individuals would be differentiated by characteristics such as insulin sensitivity quantified by the Matsuda index (Fig. 1b). Here we found a trend of the Matsuda Index correlating with the two uniform manifold approximation and projection (UMAP) dimensions using Pearson’s correlation coefficient (PCC) of 0.34 and −0.35 for dimensions one and two, respectively. Using *k*-nearest-neighbor (*k*NN) regression on the latent representation we found that *R*^2^ for Matsuda Index (*k* = 5) was 0.70 compared to 0.37–0.38 when using residualized data or dimensionality reduction using principal component analysis (PCA) and that this trend was consistent for larger *k* (Supplementary Figs. 8 and 9). This indicated that the MOVE latent representation captured a clinical signal that was not as easily identified from the residualized data or by using PCA for dimensionality reduction. Furthermore, we did not find any strong local effects of missingness (*R*^2^ = 0.05 at *k* = 5) and only small effects of age (*R*^2^ < 0.01, *k* = 100). Similarly, we used a *k*NN classifier to investigate the effect of the confounders sex and recruitment center on the global structure of the latent representation. These achieved accuracies of 0.58 and 0.25 for sex and center, respectively, which should be compared to by-chance accuracies of 0.50 and 0.17, respectively (Supplementary Figs. 10 and 11). If we used non-residualized data, that is, when not correcting for confounding effects including age, sex, and center, we observed larger effects (Supplementary Figs. 10 and 11). This demonstrates the ability of the VAE to integrate heterogeneous data but also that substantial confounding factors can influence the latent representation.

### Extracting drug to clinical and multi-omics associations

We then investigated if the model had learned associations between the clinical, drug and multi-omics data. To do this, we developed an approach that is based on perturbating input features one at a time (Fig. 1a). For instance, to identify associations between a particular drug and all other features, we simulated that we gave the drug to each of the individuals that did not receive the drug. In addition to excluding individuals that were already receiving the drug we also excluded individuals taking a drug of the same therapeutic drug-class in the anatomical therapeutic chemical classification (ATC) system (Supplementary Table 2). We then assessed if the change in each of the feature reconstructions was significantly different compared to when passing the original data through the model (Fig. 1a). Because VAE models are stochastic, we used results across an ensemble of models and developed two different approaches to identify significant associations. One approach was based on applying *t*-tests with Bonferroni correction across four different models, where each model was refitted 10 times (MOVE *t*-test), while we also, inspired by earlier variational work^29–31^, used Bayesian decision theory and a single model refitted 30 times (MOVE Bayes). To identify different parameters of the approaches that would allow for comparison across and to standard methods (*t*-test, analysis of variance (ANOVA)), we applied them to two datasets consisting of randomized clinical, drug and multi-omics data. Our findings showed that MOVE *t*-test and MOVE Bayes had good performance to identify drug–omics associations compared with *t*-test and ANOVA at a ground-truth false discovery rate (FDR) of 0.05 (Supplementary Fig. 12 and Supplementary Table 3 and Methods).

### MOVE identifies drug and multi-omics associations

We then applied the MOVE framework to identify drug associations in the DIRECT multi-modal data. The two methods, MOVE *t*-test and MOVE Bayes, identified 3,143 and 763 significant associations to the multi-omics and clinical features, respectively (Supplementary Tables 4–6 and Supplementary Data 2–4). We analyzed the intersection of the two approaches and found that 573 of the 763 (75%) of the significant associations were found by both methods (Fig. 1c). Making a conservative choice, we used the associations identified by both methods for further analyses. When compared to traditional tests such as the Student’s *t*-test and ANOVA we found this to add 211% more significant associations, from 184 to 573 (Fig. 1d). In addition, the significant associations identified by MOVE were distributed across the drugs (two-sided *t*-test, *P* = 0.016) and not only for the drugs administered to most individuals such as Simvastatin, Atorvastatin, and Metformin. For instance, MOVE identified a median of 20 associations per drug compared to 1 for *t*-test and 0 for ANOVA, highlighting that our method was more sensitive for extracting associations for drugs given to a smaller number of individuals (Supplementary Tables 5 and 6). Among the multi-omics datasets, we found that the largest number of significant drug associations was to the metabolomics, clinical, and transcriptomics data with an average of six associations per drug (Fig. 1e and Supplementary Fig. 13). When normalizing for all possible associations, the highest fraction of associations was to the clinical data (8%) followed by targeted and untargeted metabolomics with an average of 5.1% and 2.8% of the features associated to a drug, respectively. Finally, we investigated if our results could be driven by disease subtypes within the T2D cohort. To do this, we used four archetype clusters from Wesolowska–Andersen and Brorsson et al.^7^ that were based on clustering from 32 clinical features. Here we found that a median of 6.5% of the significant drug–omics associations were specific to one of the subgroups indicating that the associations were not primarily driven by the archetypes (Supplementary Table 7).

### Changes in T2D biomarkers were associated with metformin

**Fig. 2:**
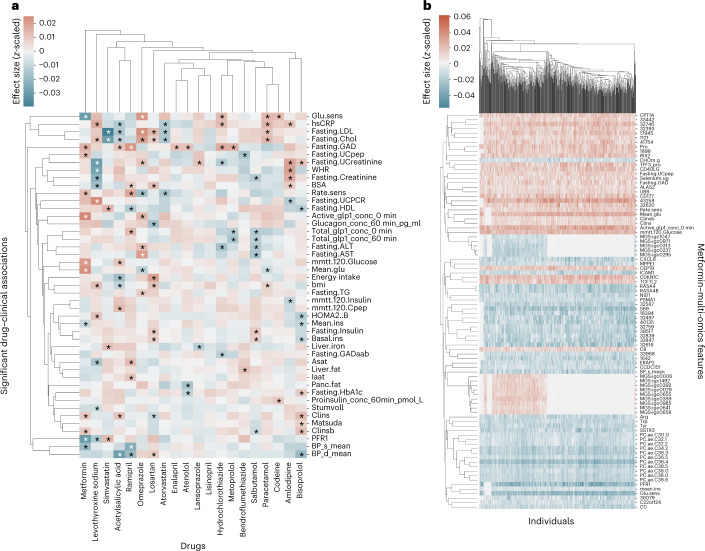
Significant associations between drugs, clinical, and multi-omics features. **a**, Significant associations between drugs and clinical features. Effects are given as effect size (*z*-scaled units) from negative (blue) to positive (red). Significant associations identified by both MOVE *t*-test and MOVE Bayes are indicated using a star. Features (*y*-axis) and drugs (*x*-axis) are clustered using hierarchical clustering on the basis of Euclidean distances. **b**, As in **a** but showing per individual-level associations of metformin to multi-omics features demonstrating that associations are highly stable across individuals. Features (*y*-axis) and newly diagnosed T2D individuals (*x*-axis). Source data

**Fig. 3:**
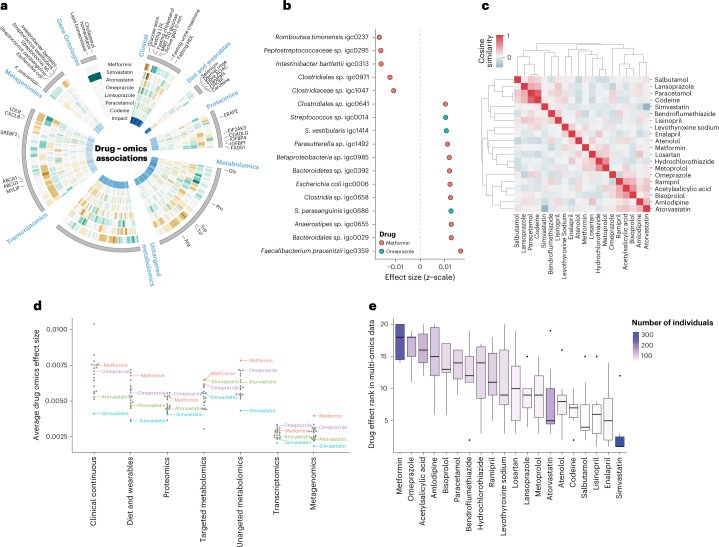
Drug associations with metagenomics species and drug–drug similarities. **a**, Display of effect sizes (*z*-scaled units) for (outer to inner) metformin, simvastatin, atorvastatin, omeprazole, lansoprazole, paracetamol, and codeine. Only significant associations to any of the drugs are shown and effect size is visualized as brown (negative), gray (none), and green (positive). Selected omics features are indicated. The Gene Ontologies element represents significantly over-represented Gene Ontology terms using transcriptomics (hypergeometric test, FDR < 0.05) (green). The innermost ring indicates SHAP importance for the individual features in the encoding from input data to the latent representation. **b**, Effect size (*z*-scaled units) (*x*-axis) of the human gut metagenomics species that were significantly associated with metformin (orange) or omeprazole (teal). **c**, Drug–drug similarities by comparing drug-response profiles across the multi-omics datasets. Cosine similarity indicated from no similarity (blue) to identical profiles (red). **d**, Average effect (*z*-score) of drugs for the omics datasets. All 20 drugs are shown, however, only metformin (red), omeprazole (purple), atorvastatin (green), and simvastatin (blue) are indicated. All other drugs are colored gray without a text label. **e**, Distribution of multi-omics ranks for the different drugs. The ranks are determined as a number between 1–20 (drugs) on the basis of the average effect size from **d**. The boxes are colored according to number of individuals taking a particular drug from 0 (white) to 323 (purple). There was no correlation between rank scores and number of individuals taking a drug (PCC = 0.14). The lower and upper hinges correspond to the first and third quartiles. The upper and lower whiskers extend from the hinge to the highest and lowest values, respectively, but no further than 1.5× interquartile range from the hinge. Data beyond the ends of whiskers are outliers and are plotted individually. Source data

We then investigated drug and multi-omics interactions (Fig. 2a and Supplementary Figs. 14–18), and initially focused on expected clinical drug interactions. For instance, for metformin, we identified 88 significant clinical and multi-omics interactions across all the datasets. When investigating associations across the individuals we found low intra-patient variability indicating that the changes were stable (Fig. 2b and Supplementary Fig. 19). We found that metformin was significantly associated with 12 clinical markers of T2D such as insulin clearance, active GLP-1, glucose levels from mixed-meal glucose tolerance test, glucose sensitivity, and blood pressure (Fig. 2a and Supplementary Data 2–4). The directions of some of the associations were opposite to the expected metformin effects, that is, metformin was associated with decreased glucose sensitivity at baseline (average *Z*-score change −0.029, confidence intervals [−0.030, −0.029]). This could be due to confounding by indication in terms of the study design where newly diagnosed T2D individuals that have been prescribed metformin are expected to have more severe clinical T2D values compared to individuals not needing medical treatments^35,36^. Therefore, since all individuals have T2D the confounding effect of their diabetic status could not be disentangled from the effect of metformin. When investigating the multi-omics associations of metformin we found two of the seven associated proteins (ERAP2 and CD40L) could be linked to the immune system (Fig. 3a and Supplementary Data 4). Similarly, for the transcriptomics data we found CXCL8 and CD177 to be altered by metformin where the former has been shown to be altered in healthy individuals and cancer patients^37–39^. In the targeted metabolomics data we identified a significant enrichment of metabolites associated with aminoacyl-tRNA biosynthesis (hypergeometric test, *P* = 2.2 × 10^−4^, FDR corrected). This pathway has previously been associated with metformin in functional pathway analysis of microbial change in mice^40^. Finally, for the untargeted metabolomics data, metformin had the highest number of associations of any drug (22 associations) indicating that new metabolic effectors of metformin treatment could potentially be identified (Supplementary Fig. 17 and Supplementary Table 4).

### Association of metformin and omeprazole with gut microbiota

Recent studies have shown how drug intake can influence the human gut microbiome composition^41,42^. Here we found metformin and omeprazole to be the only drugs to have significant associations to the metagenomics data with an increase of eleven metagenomics species as well as a decrease of six other species (Fig. 3b). Remarkably, the findings of increased *Escherichia coli* and decreased levels of *Intestinibacter bartlettii* and *Peptostreptococcaceae sp*. have been reported in healthy individuals taking metformin in an intervention study^43^ (Supplementary Data 4). As the study first reporting the findings was performed in healthy individuals, the changes are most likely not explained by other factors than metformin treatment. For omeprazole, a protein pump inhibitor (PPI), we identified three *Streptococcus* species to be significantly increased (*Streptococcus sp*., *Streptococcus parasanguinis*, and *Streptococcus vestibularis*) (Supplementary Data 4). Previous work by others has specifically shown PPIs to influence the abundance of *Streptococcus parasanguinis* and *vestibularis* in the human gut^44^. Interestingly, both omeprazole and lansoprazole target the K-transporter ATPase alpha channel 1 and increases pH in the stomach. The two drugs, however, have different speed to effect rates where omeprazole elicits its effect with a slower rate compared to lansoprazole^45^. This, in combination with more individuals being administered omeprazole (125) compared to lansoprazole (57), could explain why we identified significant alterations of gut microbiota for omeprazole and not lansoprazole.

### Statins were associated with decreased low-density lipoprotein and cholesterol

Next, we investigated associations between the two statins, simvastatin, and atorvastatin, which are widely used to treat high blood cholesterol by lowering low-density lipoprotein (LDL)^46^. In agreement with their potential to treat dyslipidemia, we found both LDL and overall cholesterol levels to be significantly associated and decreased with average LDL *z*-score change of −0.039 (CI [−0.040, −0.038]) and −0.015 (CI [−0.016, −0.014]) for simvastatin and atorvastatin, respectively (Supplementary Data 4). This effect could be a consequence of many of the participants having been administered statins before their T2D diagnosis (simvastatin median duration 1.9 years and atorvastatin median duration 1.7 years; Supplementary Table 8), thereby increasing the chance of observing the effect of the drug with reduced confounding by indication. Interestingly, we noticed that besides the downregulation of LDL and general cholesterol levels some of the remaining clinical associations were not similar. Simvastatin was associated with an increase in the health marker high-density lipoprotein (HDL) cholesterol whereas atorvastatin had a decrease. This agrees with known effects of the two statins on HDL, where simvastatin and atorvastatin, respectively, increase and decrease HDL levels with increasing doses^47^.

### Different molecular profiles of simvastatin and atorvastatin

When investigating the multi-omics associations, the two statins had diverse effects across the omics data (Fig. 3a and Supplementary Figs. 14–18 and 20). In agreement with the analysis of the clinical data, we found simvastatin to be significantly associated with downregulation of cholesterol homeostasis (Hypergeometric test, *P* = 0.005, FDR) and lipid transportation pathways (Hypergeometric test, *P* = 0.002, FDR) from the enrichment analysis of the associated transcripts (Fig. 3a and Supplementary Data 4 and 5). Specifically, we identified changes in *LDLR*, *SREBF2*, *ABCA1*, and *ABCG1* expression, previously associated with simvastatin usage and accumulation of fatty acid and triglyceride in the liver through different pathways^48–52^ (Supplementary Data 4). In the proteomics data of atorvastatin, we identified known associations to *FADS1* (ref. ^53^), as well as *EIF2AK3*, which has been reported associated with cholesterol homeostasis^54,55^. Additionally, two insulin growth factor binding proteins (*IGFBP1* and *IGFBP4*) were associated with atorvastatin and *IGFBP4* for simvastatin as well (Supplementary Data 4). These have previously been reported specifically for people with T2D and atorvastatin use^54,56^. Finally, in the targeted metabolomics data, we identified simvastatin to be associated with an increase in glycine levels, which in low systemic concentration has been associated with obesity and T2D^57^ (Supplementary Data 4). Furthermore, we observed a decrease of several phosphatidylcholines (11 of 17 decreased metabolites), and an increase of sphingomyelin and ceramide (2 of 11 increased metabolites), a ratio which has previously been shown to be altered with high doses of simvastatin compared to other statins^58^ (Supplementary Data 2–4). For atorvastatin, we observed a non-significant decrease of glycine levels and that the overall ratio of sphingomyelin and ceramide decreased (4 of 13 decreased metabolites).

### Drug polypharmacy and similarity across multi-omics data

We then investigated similarities between drugs and their multi-omics associations. Overall, we observed four clusters containing three to six drugs each and found that some of the drugs within a cluster could potentially be associated with polypharmacy (Fig. 3c). Therefore, we investigated the impact of a drug–drug combination on the associations and found a correlation between overall drug association similarity and the individuals taking the two drugs (PCC 0.75, *P* value of 2.2 × 10^−35^). This finding indicates possible polypharmacy effects introduced by taking the two drugs together resulting in a higher drug–drug similarity across all clinical and multi-omics changes. However, some of the similarities might to some extent be driven by overlapping patient groups and non-drug-related similarities such as the underlying reason for taking the drug. An example could be the drug similarity cluster of Ramipril, Acetylsalicylic Acid, Bisoprolol, Amlodipine and Atorvastatin, which can be linked to cardiovascular diseases. Furthermore, the drugs that had the most similar drug and multi-omics associations were codeine and paracetamol with a cosine similarity of 0.78. Most (38 of 46) of the individuals in the cohort taking codeine were also taking paracetamol while a large fraction of individuals (52 of 90) was only taking paracetamol. We therefore cannot rule out that the correlated multi-omics profiles of the two drugs could be driven by the partial overlap leading to similar latent representation and model reconstructions. Finally, we investigated known drug–drug interactions and association with drug multi-omics profiles; however, found no statistically significant correlations (Supplementary Note and Supplementary Fig. 21).

### The effects of drugs are widespread across the omics data

Currently, there are widespread efforts in investigating drugs and gut microbiome interactions suggesting that the microbiome is a potential target and mediator of drug effect^42,59,60^. As we investigated several multi-omics datasets besides the gut microbiome (metagenomics), we can compare the effect size of the drugs across the omics datasets. Interestingly, we found that the gut microbiome was the dataset with the second fewest number of statistically significant hits across the drugs with 17 significant associations (Supplementary Table 4 and Supplementary Fig. 13). Only diet and wearable data had fewer associations (11); transcriptomics, proteomics, targeted, and untargeted metabolomics had between 44–134 significant associations. We then asked if the effect size of the drugs were different across datasets and determined the cumulative effect size of the drugs in the respective multi-omics datasets. Here we found that the average effect sizes in transcriptomics and metagenomics data were the lowest for all drugs, and that those in the metagenomics dataset were significantly lower compared to all other omics datasets but transcriptomics (ANOVA, Tukey HSD test, adjusted *P* < 0.05) (Fig. 3d and Supplementary Table 9). When we subset to significant drug–omics associations, of which the gut microbiome only had two drugs with significant associations (metformin and omeprazole), we found that the effect of these two drugs were similar or lower compared to the effect sizes of the other multi-omics datasets (Supplementary Fig. 22). Finally, we investigated if this could be caused by increased uncertainty when learning and reconstructing a given modality but only found small correlations with PCCs of −0.15 to 0.16 between modality uncertainty and inferred effect sizes in a modality (Supplementary Table 10). Overall, this observation implies that the multi-omics response to drug stimuli are not only targeting the gut microbiome and that multiple omics datasets should be included when attempting to understand drug effects.

### Ranking the impact of drugs in multi-omics data

Finally, we investigated the effect sizes of the individual drugs across the multi-omics datasets. We found that metformin and omeprazole, in general, had the most pronounced effects on the multi-omics data (cumulative rank scores) and that the two statins ranked 14 and 20 out of the 20 drugs (Fig. 3e) where simvastatin had the lowest overall rank of cumulative effect sizes. This analysis was not confounded by the number of individuals taking a particular drug as there was no correlation (PCC = 0.14) between the number of individuals and drug effect. This was opposed to when investigating only significant associations where statins ranked 2 and 4 with high effect sizes (Supplementary Figs. 22 and 23). This observation may indicate that statins had fewer strong effects, whereas, for instance, both metformin and omeprazole with the highest average rank had larger systemic effects.

## Discussion

Here we show that it is possible to use unsupervised deep learning to integrate and extract associations from a deeply phenotyped cohort of people with T2D. While existing methods for vertical integration of multi-omics data focus on encoding the data to factors or latent representations that can be used for clustering and classification, we took this further by using the generative capacity of VAE models. In comparison to traditional univariate statistical tests, MOVE can identify significant drug–omics associations for a wider selection of drugs. We believe that these improvements come from the ability of the generative models to infer multi-omics changes for individuals not receiving a drug thus increasing power.

Previous work to stratify the newly diagnosed T2D individuals from this cohort used 32 clinical features to identify four archetypes representing different T2D subtypes^7^. In addition, they used metformin status of the individuals to investigate if the subgroups were confounded by metformin treatment and found no significant impact on the clusters and their multi-omics correlations. In contrast to their work, we added medication data on 19 additional drugs and used all data as input to our unsupervised deep-learning model allowing the model to learn from all inputs simultaneously. Thus, we were able to identify associations between the drugs and multi-omics data, including for metformin indicating the importance of vertical integration.

The cross-sectional design and clinical data-guided medical decisions make it difficult to assess the directionality of drug associations and further complicates causal inference. Hence, it is not possible to draw causal conclusions on drug effects; however, the results can be considered as input to design informed studies as well as randomized clinical control studies. In the future, expansion with longitudinal multi-omics data and modeling time could add more information on the causality of the drugs by investigating the long-term effects and associations^32^.

Similarly, our approach opens up for individualized analysis of patients in an *N*-of-1 approach^61^. It is well-known in health care that often selecting a drug or treatment in a situation at the same time excludes performing the control experiment of using another drug. Using MOVE, we can in principle ask what would happen if we gave the patient a drug and compare to the result of choosing another drug. Our cohort size is limited, but for larger cohorts of tens to hundreds of thousands of patients this could potentially be powerful to identify molecular associations and treatment outcomes for individual patients.

Finally, we emphasize that our approach is, of course, not limited to drug associations; in principle, all the omics data could be assessed for associations across the datasets. We therefore believe that our generative method opens new possibilities in big multi-omics data analysis for discoveries of potential new biomarkers, carrying out *gedankenexperiments*, and investigating potential direct effects of drugs in high dimensionality molecular data that leads to testable hypotheses.

## Methods

### The cohort

The cohort and available data included in the study are described in detail in Koivula et al.^62,63^ and Wesolowska–Andersen and Brorsson et al. (ref. ^7^). In brief, we used the newly diagnosed sub-cohort of the IMI-DIRECT study consisting of 789 participants. Fifty-eight percent of participants was male and participants had the following characteristics at baseline: age 62 (8.1) years; body mass index 30.5 (5.0) kg m^−2^; fasting glucose 7.2 (1.4) mmol l^−1^; 2 h glucose 8.6 (2.8) mmol l^−1^. Participants were diagnosed within 2 years before recruitment and had glycated hemoglobin (HbA1c) < 60.0 mmol mol^−1^ (<7.6%) within the previous 3 months. All samples represent distinct individuals. Furthermore, while Wesolowska–Andersen and Brorsson et al.^7^ used data from baseline and follow up at 18 and 36 months we only used baseline data for modeling. In addition to the baseline data from Wesolowska–Andersen and Brorsson, we carried out extensive curation and harmonization of the medication records included in the electronic case forms by the research nurses in the different recruitment centers and thus used standardized ATC annotated medication data for the individuals (see further detail below). Approval for the study protocol was obtained from each of the regional research ethics review boards separately (Lund, Sweden: 20130312105459927; Copenhagen, Denmark: H-1-2012-166 and H-1-2012-100; Amsterdam, Netherlands: NL40099.029.12; Newcastle, Dundee, and Exeter, UK: 12/NE/0132) and all participants provided written informed consent at enrollment. The research conformed to the ethical principles for medical research involving human participants outlined in the declaration of Helsinki. Further details about the data generation can be found in Wesolowska–Andersen and Brorsson et al.^7^.

### Pre-processing of data

From the clinical, environmental, and questionnaire data only variables with variation across the dataset that were present in at least 10% of the individuals were included. The genomic data was included as the genotypes of risk alleles identified in Mahajan et al.^64^. In total 393 risk alleles were identified in our cohort out of the 403 associations mentioned in the paper. The genotypes were included as homozygous for risk allele, heterozygote, not having the allele, or missing if the locus was not identified for the individual. Diet data was included as 47 features on self-reported total intake of macronutrients and vitamins across a 24-h period. The wearables measured with an accelerometer included 25 measurements that summarize the movement and heart rate during the day. Transcriptomics data (RNA sequencing) from fasting whole blood samples were processed with RailRNA (v0.2.4b)^65^ to obtain scaled counts for all samples and only the most variable genes were included. The variable genes were selected by calculating the standard deviation across all individuals for each gene and selecting genes with an above-average standard deviation. Both targeted and untargeted metabolomics data in fasting plasma were included for all measurements passing quality control. In the proteomics data, all measurements within the measurable range based on the OLINK antibody panel were included and residualized for plate layout. The metagenomics data was only available for approximately one-third (256) of the individuals and were included as normalized read counts of identified Metagenomic Species^66^. Categorical data, including questionnaire responses, drug data, and genomics, was one-hot encoded. The continuous data were residualized by the collection center as the data was collected from six different European countries and, thus, handled by different nurses and lab technicians, as well as differences in the time-of-day samples were taken, which could have a large effect on the measurements. Additionally, the data were residualized for age and sex as these could be biological non-disease-related confounders in the data. Lastly, each continuous dataset was *z*-scale normalized per feature to ensure that each feature was distributed around zero.

### Classification of drugs using the ATC system

The ATC system is the WHO classification system for therapeutic drugs. The system has a hierarchical structure, where the topmost level*,* ‘level 1—Anatomical main group’, specifies the target organ or tissue, and the lowermost level, ‘level 5—chemical substance’, specifies the active chemical compound. The three levels in between specify the therapeutic, pharmacological, and chemical levels, respectively. We, therefore, mapped all drugs to the lowest possible level to prevent information loss. A total of 4,155 entries could be mapped to level 5. For 55 entries, only a higher-level mapping was possible owing to lack of specificity and 43 entries could not be mapped to the ATC system, either because of the compound not existing in the database, for example nutraceutical compounds, or when we were unable to identify which drug was registered for the participant. The ATC system does not only specify compound names, but also administration route and daily dosages for over half of level 5 entries. However, owing to uncertainty of the reliability of the registered dosages, only drug names and administration routes were used for mapping. In instances where the administration route was not available, the drug was mapped by drug name only.

### Drug data collection and clean-up

The study participants were asked to register their current drug usage at screening and baseline. Drug names were registered as free text together with administration route, dosage and frequency, and indication. Metformin was recorded separately from other anti-diabetic and non-anti-diabetic drugs. The collected data was variable in quality, using both generic and brand names, which were in many cases specific to the country of the participant. The data was cleaned in four steps: (1) removal of special characters, company names, formulations, and other non-relevant information; (2) automatic mapping to the PubChem database; (3) manual mapping to generic drug names; and (4) mapping to the ATC system. Indications of placebo use, for example participation in clinical drug trials, were noted as such. Only active compounds were included and consequently, possible brand variation was ignored, including for dietary supplements. Drug combinations were mapped, when possible, to the ATC code specifying said combination. However, when the specificity of the proposed ATC code was less specific than the registered drugs, the drug combinations were mapped to individual ATC codes, that is, ‘Perindopril’ (C09AA04) and ‘Indapamide’ (C03BA11) was used instead of ‘Perindopril and diuretics’ (C09BA04). Entries were mapped to ATC codes with the administration route when possible and otherwise mapped without the administration route. Dosage information was not used in the mapping process. In the manual mapping process, 99.4% of terms were assigned and a total of 359 drugs and drug combinations were identified. A total of 339 drugs (94.4%) was mapped to 441 ATC codes.

### Design of the VAE

The VAE framework was constructed to account for a variable number of fully connected hidden layers in both the encoder and decoder and a latent layer that samples from a Gaussian distribution N(0, 1) of two vectors of size *N*_*L*_ representing the means, *µ*, and standard deviations, *σ*. Each hidden layer included both batch normalization and dropout^67^ and with leaky rectified linear units (LeakyReLU)^68^ as activation function. Each dataset was concatenated to one input layer of both categorical and continuous variables. To allow for dataset-specific weights the error calculation was done separately for each dataset. Here we applied cross-entropy loss for categorical data and mean squared error for continuous data as implemented in PyTorch^69^. The loss was normalized by dataset input size and batch size. Deviance from the Gaussian distribution was penalized by adding the Kullback–Leibler divergence (KLD) to the loss. The final loss was defined as

$$L = \mathbf{W}_{\mathrm{cat}} \times \mathbf{E}_{\mathrm{cat}} + \mathbf{W}_{\mathrm{con}} \times \mathbf{E}_{\mathrm{con}} + \mathbf{W}_{\mathrm{KLD}} \times \mathrm{KLD}$$

Here, **E**_cat_ and **E**_con_ are vectors of normalized reconstruction error for each of the continuous and categorical datasets. **W**_cat_ and **W**_con_ are vectors as well of the same length as the errors to introduce dataset-specific weights. We applied an equal weight of 1 for all datasets except for continuous clinical data where we used a weight of 2. **W**_KLD_ is a weight put on the KLD defined as **W**_KLD_ = *β* × *N*_*L*_^*−1*^ for which we used a *β* of 0.0001 for the final model. The KLD was defined as

$$\mathrm{KLD} = {\sum} { - \frac{1}{2}(1 + \ln \left( \sigma \right) - \mu ^2 - \sigma )}$$

To efficiently handle missing data for the continuous features we encoded them as mean values across a particular feature during training and excluded the missing data points during back-propagation. With the data being *z*-score normalized the mean value is represented as zero. For the categorical features, we included them as a zero vector and the ignore index feature in the cross-entropy implementation in PyTorch was used to not include errors for missing data in the back-propagation. The VAE model was trained with the Adam optimizer^70^, with a mini-batch size of 10 and increasing batch size with a factor of 1.25 during training after every 50 epochs. The number of training epochs was set to 200 on the basis of early stopping on the test set as described below. Additionally, we trained the model using warm-up by first including the full KLD after 10 epochs slowly increasing the weight at epochs 4, 6, and 8. The latent representation of each patient was obtained by passing them through the trained VAE and extracting the *µ* layer. The VAE was implemented using PyTorch^69^ (v.1.7.0) and run using a GPU running CUDA (v.10.2.89).

### Hyperparameter optimization for multi-omics integration

We initially divided the dataset into training (90%) and test (10%) sets to identify the optimal hyperparameter settings to efficiently capture the data structure without losing the ability to generalize on the test data (Supplementary Figs. 2 and 3). We tested different combinations of sizes of hidden layers, the number of hidden layers, size of latent space, dropout, and weight on the KLD. We then evaluated the model on the basis of both test log-likelihood and reconstruction accuracy. For the number of hidden neurons, the variations used were 200, 500, 800, 1,000, and 1,200, with the number of layers ranging between 1 and 5. The tested latent sizes were between 20 and 400 as well as dropout of 10%, 20%, and 30% and KLD weights of 0.001, 0.0001, and 0.0001. We defined an accurate reconstruction for categorical variables as the class with the highest probability corresponding to the class given by the input. For continuous variables, the accuracy was assessed by comparing the reconstructed array with the input array using cosine similarity for each individual instead of using exact matching. For both categorical and continuous data only non-missing values were used when calculating the accuracy in the reconstruction. We chose the number of training epochs on the basis of when the optimal test likelihood was achieved during testing rounded up to the nearest 100 epochs to ensure sufficient training to learn the complexity of the data. Here we found that more complex models, with higher numbers of hidden neurons and layers, resulted in worse performance on the test set (Supplementary Fig. 2) and that models with more than one hidden layer were unable to provide a decent reconstruction on the training data without overfitting. The only exception was the size of the latent representation, which gave a worse performance with smaller sizes (<50) and equally good performance for larger sizes (from 100 to 400) (Supplementary Fig. 3). For the five best performing models, stability was measured to choose the final model. The stability of the model was evaluated by repeating training with the same hyperparameters and calculating the difference in cosine similarity of the latent space to all other individuals. If the model produced the same result the average change in cosine similarity should be zero. The model with the average change closest to zero was then considered the most stable. The final hyperparameters were set to be one hidden layer of 2,000 neurons, a latent size of at least 100, and a 10% dropout for regularization.

### Evaluating feature importance

Feature importance was extracted from the weights of the network for the models with only one hidden layer and because the input data was *z*-score normalized calculated as

$$I_i = \mathop {\sum }\limits_{j = 1}^{n_{\mathrm{hidden}}} \left| {w_{ij}} \right|$$

where *I*_*i*_ is the *i*th feature input and $\left| {w_{ij}} \right|$ is the absolute value of the weight from *i*th input to the *j*th hidden neuron. To assess the actual impact on the latent representation an adaptation of the SHAP^19^ analysis was applied. The difference in model performance was assessed as the absolute differences of the latent representation when changing each input to missing for all individuals and passing it through the trained model.

### Extracting significant drug associations

Drug associations were extracted by perturbation of the input data after training the final model on all individuals. Thus, for each drug we changed the drug status for all individuals with ‘not receiving’ to ‘receiving’. Importantly, we only included individuals that did not receive the specific drug or another drug within the same therapeutic subgroup (ATC level 2). Then, for each drug change, we compared the change in reconstructions to when we passed the original (un-perturbed) data through the network. In other words we determined the differences that the network infers from the change in drug status that during training was learned from all individuals receiving the drug. We used two strategies for this, one was based on an ensemble of Student’s *t*-tests using benchmarked thresholds, and another was based on Bayesian decision theory. Both approaches were benchmarked against randomized datasets where all the input data matrices were shuffled on rows and columns. We simulated effects in the shuffled data by randomly sampling a combination of a drug, a multi-omics dataset, and a feature within that omics dataset. For each combination, we then sampled an effect from the standard normal distribution N(0,1) and added this value to the omics feature whenever the selected drug was taken by an individual. We, therefore, did not expect that all effects would be significant in the statistical tests because we sample from N(0,1) and some effects will be close to 0. We added a total of 100 effects to the shuffled data and repeated the entire procedure to generate two shuffled datasets each with their unique added effects. Additionally, we investigated if the number of significant associations, effect size estimates and model uncertainty in the reconstruction were not biased by individual dataset uncertainties. This was done by calculating PCCs between the average estimated effect size across all 20 drugs and the difference between model input and the reconstructions for each of the omics features.

### Significant associations using MOVE *t*-test

To evaluate if the change in the reconstruction was significant, we first determined the expected average change when passing the original and perturbed data through the model ten times. On the basis of these averages, we used a Student’s *t*-test for related samples as implemented in Python SciPy (v.1.3.1)^71^ between the baseline and drug-perturbed data for all non-missing continuous data. All *P* values were subsequently Bonferroni-corrected independently for each drug, and we applied a significance threshold of adjusted *P* < 0.05. We repeated the entire analysis with retraining of the model 10 times for each of four latent sizes (150, 200, 250, and 300). Associations were only included for analysis if they were significant for at least three of the four latent sizes and in at least five out of ten of the repeats. Therefore, reported *P* values were the averaged *P* value across the 10 replicate and 4 model tests, that is a total of 40 two-sided Bonferroni-corrected *t*-tests. The change in reconstruction, what we report as effect size, was calculated as the average difference across the 10 replicates and 4 model tests and were reported with 95% confidence intervals.

### Significant associations using Bayes decision theory

For the method that was based on Bayesian decision theory we used an approach inspired by single-cell variational inference^29^ and Lopez et al.^31^. We trained VAE models with a latent size of 150 neurons and benchmarked the approach using different latent sizes and ensembling 1, 5, 10, 20, 30, 35, 40, or 50 models, which we termed refits. For the refits we averaged the reconstructions and used these to obtain the posteriors for the non-perturbed data and each of the drug perturbations. Thus, for VAE ensemble refit *i*, individual *n*, feature *f*, and drug *d* we define the variational reconstructions as $\hat x_{infd}$. By averaging across VAE refits, we obtain estimates of the average posteriors $\hat x_{nfd}$. Then, for each drug *d* we compare between two models: $M_d^f$ where feature *f* is significantly associated with the drug, and the alternative model $M_0^f$ where feature *f* is not significantly associated with drug *d*. Hence, we evaluate how often $\left| {\hat x_{nfd} - \hat x_{nf0}} \right| > 0$ and calculate Bayes factors (*K*) as:

$$K = {{{\mathrm{log}}}}_e\left| {\frac{{\mathrm{P}\left( {M_d^f|\hat x_{fd},\,\hat x_{f0}} \right)}}{{\mathrm{P}(M_0^f|\hat x_{fd},\,\hat x_{f0})}}} \right|$$

We ranked the associated features according to *K* (ref. ^72^). We set a FDR of *α* by accepting associations (*n*) between features and a drug until the cumulative evidence of P(*M*_0_) across accepted features for the drug was above the threshold. Since $\mathrm{P}(M_0^f)=(1-\mathrm{P}(M_d^f))$ we accepted drug-feature associations while the cumulative evidence *E* is lower than α

$$E = \mathop {\sum }\limits_f \frac{{(1 - \mathrm{P}(M_d^f))}}{n} < \alpha$$

### Benchmarking of *t*-test, MOVE *t*-test and MOVE Bayes

To be able to compare the number of significant associations between methods we used the two randomized datasets to estimate FDR from the ground truth, that is the added drug–omics effects (Supplementary Table 3). Here we found that a *t*-test with Benjamini–Hochberg FDR of 0.01 had ground-truth FDR of 0.00 and 0.06 on the two randomized datasets, corresponding to 52 and 67 true positives as well as 0 and 4 false positives, respectively. For MOVE *t*-test, we benchmarked the number of refits of the 4 models and found 10 refits to have a ground-truth FDR of 0.02 and 0.06, with 48 and 61 true positives as well as 1 and 3 false positives, respectively. For MOVE Bayes we benchmarked the number of refits for a model with 150 latent neurons and found FDR from the cumulative evidence to be well aligned with FDR of the ground truth. Using Bayes FDR of 0.05 we found 30 refits to have ground-truth FDR of 0.02 and 0.05, respectively. Across the two shuffled datasets 42 and 59 true positives were found by all three methods (Supplementary Fig. 12).

### Calculation of drug associations using other methods

We compared our findings to associations identified with standard statistical approaches using Student’s *t*-test for unrelated samples and an ANOVA between two groups of individuals ‘not receiving’ and ‘receiving’ each drug. Here we used Benjamini–Hochberg correction for FDR^73^ with an adjusted *P* < 0.01. Additionally, we tested if a least absolute shrinkage and selection operator (LASSO) model was able to identify features with significant impact on predicting the ‘not receiving’ or ‘receiving’ groups for each drug. However, the LASSO model was unable to converge possibly owing to the high input feature dimensionality. All statistical tests were done with Python SciPy (v.1.3.1)^71^.

### Drug effect size and similarities across omics data

Drug effect sizes were determined as the difference between the baseline and drug-perturbed variational reconstructions, that is, as the average difference across the VAE ensemble refits reported with 95% confidence intervals. Drug similarities were calculated as the cosine similarity as implemented in Python SciPy (v.1.3.1)^71^ between the average effect sizes on all features identified as significantly associated for at least one of the drugs both across and within each dataset. The difference was only calculated for non-missing data and individuals not already on the drug or a drug in the same ACT group. The rank of drug effect sizes was determined for each omics dataset ranking the effect sizes from 1 to 20. A rank of 20 indicates that the drug had the highest average effect size in this omics dataset compared to the other drugs. Correlations between multi-omics profiles and number of individuals taking the drug pair were calculated from the fraction of individuals that overlapped between the two drugs.

### Molecular-focused analysis of the multi-omics data

To get a better understanding of the molecular profiles identified in the associations for the transcriptomics and proteomics data we tested for enriched Gene Ontology terms as well as molecular pathways. For the transcriptomics data, we assessed the molecular patterns of biological processes and pathways from Reactome^74^ (v.3.7) using the significantly associated genes for each drug against a background list of all genes included in the data integration. We used WebGestaltR^75^ (v.0.4.4) for the analysis with default settings (hypergeometric test) and evaluated all results with an FDR < 0.05. The targeted metabolomics data was analyzed for potential metabolite enrichments using MetaboAnalyst^76^ (v.5) over-representation analysis using a hypergeometric test and FDR of 0.05. We investigated both enrichments in known pathways in the KEGG database as well as enrichment of chemical structures sub-, main- and super-class levels. For all analyses, we used the included panel of targeted metabolites as the reference data.

### Association differences within diabetes archetypes

As mentioned, previous work by Wesolowska–Andersen and Brorsson et al. performed archetype analysis of the multi-omics data with only metformin medication data^7^. Here they based the archetypes on clinical markers and identified four distinct and one ‘mixed’ T2D archetypes with clinical and omics profiles. To investigate if these distinct archetypes differed in their drug associations we used a *t*-test on the average effect size change for the individuals of each archetype against the remaining individuals. The analysis was only done for the significant drug associations for each drug. All analysis was only done for individuals not taking the drug or a drug within the same ATC therapeutical class similarly to the main analysis.

### Drug–drug interactions

We used an in-house drug–drug interaction compendium generated from publicly available sources (Supplementary Table 11) to assess whether drug combinations had been reported previously to be interacting or not^77^. The compendium contains interactions from 26 different datasets of pharmacovigilance, clinically oriented information, schemas for NLP corpora, and drug–Cytochrome P450 relationships sources. For 12 of the drug–drug pairs in our dataset we could identify drug–drug interactions with reported severity (major, moderate, minor, possible, undetermined, and none) indicating clinical significance.

### Reporting summary

Further information on research design is available in the Nature Research Reporting Summary linked to this article.

## Online content

Any methods, additional references, Nature Research reporting summaries, source data, extended data, supplementary information, acknowledgements, peer review information; details of author contributions and competing interests; and statements of data and code availability are available at 10.1038/s41587-022-01520-x.

## Supplementary information

Supplementary Information Supplementary Notes, Supplementary Figs. 1–23, and Supplementary Tables 1–11.

Reporting Summary

Supplementary Data 1 Features that were included from the different omics datasets.

Supplementary Data 2 MOVE *t*-test, adjusted *P* values and effect sizes.

Supplementary Data 3 MOVE Bayes, adjusted *P* values.

Supplementary Data 4 Significant associations in both MOVE *t*-test and MOVE Bayes.

Supplementary Data 5 Over-representation analysis using Gene Ontologies.

## Data Availability

Owing to the informed consent given by study participants, the various national ethical approvals for the present study, and the European General Data Protection Regulation (GDPR), individual-level clinical and omics data cannot be transferred from the centralized IMI-DIRECT repository. Requests for access to summary statistics of the IMI-DIRECT data, including those presented here, can be made to directdataaccess@dundee.ac.uk. Requesters will be informed on how summary-level data can be accessed via the DIRECT secure analysis platform following submission of an appropriate application. The IMI-DIRECT data access policy is available at https://directdiabetes.org. Example data is available at https://github.com/RasmussenLab/MOVE/ for testing of MOVE. As described in the methods section we used ATC (https://www.who.int/tools/atc-ddd-toolkit/atc-classification) and WebGestalt (v.0.4.4 at http://www.webgestalt.org) for analysis of Gene Ontologies, Reactome (v.3.7 at https://reactome.org) for analysis of molecular pathways, and MetaboAnalyst (v.5 at https://www.metaboanalyst.ca) for analysis of targeted metabolomics data. The 25 databases of drug–drug interactions are listed in Supplementary Table 11. Source data are provided with this paper.

## Source data

Source Data Fig. 1 Source data for Fig. 1c–e.
Source Data Fig. 2 Source data for the Fig. 2a.
Source Data Fig. 3 Source data for Fig. 3a–e.

## Code availability

The MOVE pipeline is available at https://github.com/RasmussenLab/MOVE/.
