# Author Correction: Discovery of drug–omics associations in type 2 diabetes with generative deep-learning models

**DOI:** 10.1038/s41587-023-01805-9

**Published:** 2023-05-02

**Authors:** Rosa Lundbye Allesøe, Agnete Troen Lundgaard, Ricardo Hernández Medina, Alejandro Aguayo-Orozco, Joachim Johansen, Jakob Nybo Nissen, Caroline Brorsson, Gianluca Mazzoni, Lili Niu, Jorge Hernansanz Biel, Cristina Leal Rodríguez, Valentas Brasas, Henry Webel, Michael Eriksen Benros, Anders Gorm Pedersen, Piotr Jaroslaw Chmura, Ulrik Plesner Jacobsen, Andrea Mari, Robert Koivula, Anubha Mahajan, Ana Vinuela, Juan Fernandez Tajes, Sapna Sharma, Mark Haid, Mun-Gwan Hong, Petra B. Musholt, Federico De Masi, Josef Vogt, Helle Krogh Pedersen, Valborg Gudmundsdottir, Angus Jones, Gwen Kennedy, Jimmy Bell, E. Louise Thomas, Gary Frost, Henrik Thomsen, Elizaveta Hansen, Tue Haldor Hansen, Henrik Vestergaard, Mirthe Muilwijk, Marieke T. Blom, Leen M. ‘t Hart, Francois Pattou, Violeta Raverdy, Soren Brage, Tarja Kokkola, Alison Heggie, Donna McEvoy, Miranda Mourby, Jane Kaye, Andrew Hattersley, Timothy McDonald, Martin Ridderstråle, Mark Walker, Ian Forgie, Giuseppe N. Giordano, Imre Pavo, Hartmut Ruetten, Oluf Pedersen, Torben Hansen, Emmanouil Dermitzakis, Paul W. Franks, Jochen M. Schwenk, Jerzy Adamski, Mark I. McCarthy, Ewan Pearson, Karina Banasik, Simon Rasmussen, Søren Brunak, IMI DIRECT Consortium, Philippe Froguel, Cecilia Engel Thomas, Ragna Haussler, Joline Beulens, Femke Rutters, Giel Nijpels, Sabine van Oort, Lenka Groeneveld, Petra Elders, Toni Giorgino, Marianne Rodriquez, Rachel Nice, Mandy Perry, Susanna Bianzano, Ulrike Graefe-Mody, Anita Hennige, Rolf Grempler, Patrick Baum, Hans-Henrik Stærfeldt, Nisha Shah, Harriet Teare, Beate Ehrhardt, Joachim Tillner, Christiane Dings, Thorsten Lehr, Nina Scherer, Iryna Sihinevich, Louise Cabrelli, Heather Loftus, Roberto Bizzotto, Andrea Tura, Koen Dekkers, Nienke van Leeuwen, Leif Groop, Roderick Slieker, Anna Ramisch, Christopher Jennison, Ian McVittie, Francesca Frau, Birgit Steckel-Hamann, Kofi Adragni, Melissa Thomas, Naeimeh Atabaki Pasdar, Hugo Fitipaldi, Azra Kurbasic, Pascal Mutie, Hugo Pomares-Millan, Amelie Bonnefond, Mickael Canouil, Robert Caiazzo, Helene Verkindt, Reinhard Holl, Teemu Kuulasmaa, Harshal Deshmukh, Henna Cederberg, Markku Laakso, Jagadish Vangipurapu, Matilda Dale, Barbara Thorand, Claudia Nicolay, Andreas Fritsche, Anita Hill, Michelle Hudson, Claire Thorne, Kristine Allin, Manimozhiyan Arumugam, Anna Jonsson, Line Engelbrechtsen, Annemette Forman, Avirup Dutta, Nadja Sondertoft, Yong Fan, Stephen Gough, Neil Robertson, Nicky McRobert, Agata Wesolowska-Andersen, Andrew Brown, David Davtian, Adem Dawed, Louise Donnelly, Colin Palmer, Margaret White, Jorge Ferrer, Brandon Whitcher, Anna Artati, Cornelia Prehn, Jonathan Adam, Harald Grallert, Ramneek Gupta, Peter Wad Sackett, Birgitte Nilsson, Konstantinos Tsirigos, Rebeca Eriksen, Bernd Jablonka, Mathias Uhlen, Johann Gassenhuber, Tania Baltauss, Nathalie de Preville, Maria Klintenberg, Moustafa Abdalla

**Affiliations:** 1 Novo Nordisk Foundation Center for Protein Research, Faculty of Health and Medical Sciences, University of Copenhagen, Copenhagen, Denmark; 2 Department of Health Technology, Technical University of Denmark, Kongens Lyngby, Denmark; 3 Copenhagen Research Centre for Mental Health, Mental Health Centre Copenhagen, Copenhagen University Hospital, Copenhagen, Denmark; 4 Department of Immunology and Microbiology, Faculty of Health and Medical Sciences, University of Copenhagen, Copenhagen, Denmark; 5 C.N.R. Institute of Neuroscience, Padova, Italy; 6 Wellcome Centre for Human Genetics, University of Oxford, Oxford, UK; 7 Department of Genetic Medicine and Development, University of Geneva Medical School, Geneva, Switzerland; 8 Biosciences Institute, Faculty of Medical Sciences, Newcastle University, Newcastle, UK; 9 Research Unit of Molecular Epidemiology, Helmholtz Zentrum München, German Research Center for Environmental Health, Neuherberg, Bavaria Germany; 10 Institute of Epidemiology, Helmholtz Zentrum München, German Research Center for Environmental Health, Neuherberg, Bavaria Germany; 11 Chair of Food Chemistry and Molecular and Sensory Science, Technical University of Munich, Freising, Germany; 12 Metabolomics and Proteomics Core, Helmholtz Zentrum Muenchen, German Research Center for Environmental Health, Neuherberg, Germany; 13 Affinity Proteomics, Science for Life Laboratory, School of Engineering Sciences in Chemistry, Biotechnology and Health, KTH Royal Institute of Technology, Solna, Sweden; 14 Research and Development Global Development, Translational Medicine and Clinical Pharmacology, Sanofi-Aventis Deutschland, Frankfurt, Germany; 15 Novo Nordisk Foundation Center for Basic Metabolic Research, Faculty of Health and Medical Sciences, University of Copenhagen, Copenhagen, Denmark; 16 University of Exeter Medical School, Exeter, UK; 17 The Immunoassay Biomarker Core Laboratory, School of Medicine, University of Dundee, Dundee, UK; 18 Research Centre for Optimal Health, Department of Life Sciences, University of Westminster, London, UK; 19 Section for Nutrition Research, Faculty of Medicine, Imperial College London, London, UK; 20 Department of Radiology, Copenhagen University Hospital Herlev-Gentofte, Herlev, Denmark; 21 Department of Epidemiology and Data Science, Amsterdam Public Health Research Institute, Amsterdam UMC, Vrije Universiteit Amsterdam, Amsterdam, the Netherlands; 22 Department of General Practice, Amsterdam Public Health Research Institute, Amsterdam UMC, Vrije Universiteit Amsterdam, Amsterdam, the Netherlands; 23 Department of Biomedical Data Science, Section Molecular Epidemiology, Leiden University Medical Center, Leiden, the Netherlands; 24 Department of Cell and Chemical Biology, Leiden University Medical Center, Leiden, the Netherlands; 25 Inserm, Univ Lille, CHU Lille, Lille Pasteur Institute, EGID, Lille, France; 26 MRC Epidemiology Unit, University of Cambridge School of Clinical Medicine, Cambridge, UK; 27 Department of Medicine, University of Eastern Finland, Kuopio, Finland; 28 Institute of Cellular Medicine, Newcastle University, Newcastle, UK; 29 Diabetes Research Network, Royal Victoria Infirmary, Newcastle, UK; 30 Centre for Health, Law and Emerging Technologies (HeLEX), Faculty of Law, University of Oxford, Oxford, UK; 31 Lund University Diabetes Centre, Department of Clinical Sciences, Lund University, Malmö, Sweden; 32 Translational and Clinical Research Institute, Faculty of Medical Sciences, Newcastle University, Newcastle, UK; 33 Division of Population Health & Genomics, School of Medicine, University of Dundee, Dundee, UK; 34 Genetic and Molecular Epidemiology Unit, Lund University Diabetes Centre, Department of Clinical Sciences, CRC, Lund University, SUS, Malmö, Sweden; 35 Eli Lilly Regional Operations, Vienna, Austria; 36 Harvard T.H. Chan School of Public Health, Boston, MA USA; 37 OCDEM, Radcliffe Department of Medicine, University of Oxford, Oxford, UK; 38 Institute of Experimental Genetics, Helmholtz Zentrum München, German Research Center for Environmental Health, Neuherberg, Germany; 39 Department of Biochemistry, Yong Loo Lin School of Medicine, National University of Singapore, Singapore, Singapore; 40 Institute of Biochemistry, Faculty of Medicine, University of Ljubljana, Ljubljana, Slovenia; 41 Oxford Centre for Diabetes, Endocrinology and Metabolism, University of Oxford, Oxford, UK; 42 Present Address: Genentech, South San Francisco, CA USA; 43 Department of Metabolism, Digestion and Reproduction, Imperial College London, London, UK; 44 Biophysics Institute (IBF-CNR), National Research Council of Italy, Milan, Italy; 45 Department of Biosciences, University of Milan, Milan, Italy; 46 Biotech & Biomarkers Research Department, Institut de Recherches Internationales Servier, Croissy sur Seine, France; 47 Blood Sciences, Royal Devon and Exeter NHS Foundation Trust, Exeter, UK; 48 Boehringer Ingelheim International, Therapeutic Area CardioMetabolism and Respiratory Medicine, Ingelheim am Rhein, Germany; 49 Boehringer Ingelheim International, Therapeutic Area CNS, Retinopathies and Emerging Areas, Ingelheim am Rhein, Germany; 50 Boehringer Ingelheim International, Medicine Cardiometabolism and Respiratory, Biberach an der Riss, Germany; 51 Boehringer Ingelheim International, Translational Medicine & Clinical Pharmacology, Biberach an der Riss, Germany; 52 Centre for Mathematics and Algorithms for Data, University of Bath, Bath, UK; 53 Clinical Operations, Sanofi-Aventis Deutschland, Frankfurt, Germany; 54 Clinical Pharmacy, Saarland University, Saarbrücken, Germany; 55 Clinical Research Centre, Ninewells Hospital and Medical School, University of Dundee, Dundee, Scotland UK; 56 Department of Mathematical Sciences, University of Bath, Bath, UK; 57 Digital and Data Sciences, Sanofi-Aventis Deutschland, Frankfurt, Germany; 58 Eli Lilly and Company, Indianapolis, IN USA; 59 Institute for Epidemiology and Medical Biometry, ZIBMT, University of Ulm, Ulm, Germany; 60 Institute of Biomedicine, Bioinformatics Center, University of Eastern Finland, Kuopio, Finland; 61 Institute of Clinical Medicine, Internal Medicine, University of Eastern Finland, Kuopio, Finland; 62 German Center for Diabetes Research, München-Neuherberg, Germany; 63 Lilly Deutschland, Bad Homburg, Germany; 64 Medizinische Universitätsklinik Tübingen, Eberhard Karls Universität Tübingen, Tübingen, Germany; 65 NIHR Exeter Clinical Research Facility, University of Exeter Medical School, Exeter, UK; 66 Regulatory Genomics and Diabetes, Centre for Genomic Regulation, CIBERDEM, Barcelona, Spain; 67 Strategy and Innovation, Sanofi-Aventis Deutschland, Frankfurt, Germany; 68 Systems Biology, Science for Life Laboratory, School of Engineering Sciences in Chemistry, Biotechnology and Health, KTH Royal Institute of Technology, Solna, Sweden; 69 TMED, Sanofi-Aventis Deutschland, Frankfurt, Germany; 70 Translational and Clinical Research, Metabolism Innovation Pole, Institut de Recherches Internationales Servier, Suresnes Cedex, France

**Keywords:** Data integration, Systems biology, Type 2 diabetes, Machine learning

Correction to: *Nature Biotechnology* 10.1038/s41587-022-01520-x. Published online 2 January 2023.

In the version of this article initially published, Cristina Leal Rodríguez (Novo Nordisk Foundation Center for Protein Research, Faculty of Health and Medical Sciences, University of Copenhagen, Copenhagen, Denmark) was omitted from the author list. The error has been corrected in the HTML and PDF versions of the article.

