# Supplementary material for: Discovery of drug–omics associations in type 2 diabetes with generative deep-learning models
Source: Nat Biotechnol. 2023 Jan 2;41(3):399–408. doi: 10.1038/s41587-022-01520-x (PMC10017515; doi:10.1038/s41587-022-01520-x)
Supplement: Supplementary file 1 — Supplementary Notes, Supplementary Figs. 1–23, and Supplementary Tables 1–11. [file 41587_2022_1520_MOESM1_ESM.pdf]

nature biotechnology

Article https://doi.org/10.1038/s41587-022-01520-x

Discovery of drug–omics associations in type 2 diabetes with generative deep-learning models

In the format provided by the authors and unedited

Supplementary Information

Discovery of drug-omics associations in type 2 diabetes with generative deep-learning models

Author list

Rosa Lundbye Allesøe[1,2,3], Agnete Troen Lundgaard[1,2], Ricardo Hernández Medina[1], Alejandro Aguayo-Orozco[1,2], Joachim Johansen[1,2], Jakob Nybo Nissen[1], Caroline Brorsson[1,2], Gianluca Mazzoni[1,2], Lili Niu[1], Jorge Hernansanz Biel[1,2], Valentas Brasas[1], Henry Webel[1], Michael Eriksen Benros[3,4], Anders Gorm Pedersen[2], Piotr Jaroslaw Chmura[1,2], Ulrik Plesner Jacobsen[1,2], Andrea Mari[5], Robert Koivula[6], Anubha Mahajan[6], Ana Vinuela[7,8], Juan Fernandez Tajes[6], Sapna Sharma[9,10,11], Mark Haid[12], Mun-Gwan Hong[13], Petra B. Musholt[14], Federico De Masi[1,2], Josef Vogt[15], Helle Krogh Pedersen[2,15], Valborg Gudmundsdottir[1,2], Angus Jones[16], Gwen Kennedy[17], Jimmy Bell[18], E. Louise Thomas[18], Gary Frost[19], Henrik Thomsen[20], Elizaveta Hansen[20], Tue Haldor Hansen[15], Henrik Vestergaard[15], Mirthe Muilwijk[21], Marieke T. Blom[22], Leen M. 't Hart[21,23,24], Francois Pattou[25], Violeta Raverdy[25], Soren Brage[26], Tarja Kokkola[27], Alison Heggie[28], Donna McEvoy[29], Miranda Mourby[30], Jane Kaye[30], Andrew Hattersley[16], Timothy McDonald[16], Martin Ridderstråle[31], Mark Walker[32], Ian Forgie[33], Giuseppe N. Giordano[34], Imre Pavo[35], Hartmut Ruetten[14], Oluf Pedersen[15], Torben Hansen[15], Emmanouil Dermitzakis[7], Paul W. Franks[31,36,37], Jochen M. Schwenk[13], Jerzy Adamski[38,39,40], Mark I. McCarthy[6,41,42], Ewan Pearson[33], Karina Banasik[1,2], Simon Rasmussen[1], Søren Brunak[1,2], IMI DIRECT Consortium

Affiliations

1 Novo Nordisk Foundation Center for Protein Research, Faculty of Health and Medical Sciences, University of Copenhagen, Copenhagen, Denmark

2 Department of Health Technology, Technical University of Denmark, Kongens Lyngby, Denmark

3 Copenhagen Research Centre for Mental Health, Mental Health Centre Copenhagen, Copenhagen University Hospital, Denmark

4 Department of Immunology and Microbiology, Faculty of Health and Medical Sciences, University of Copenhagen, Copenhagen, Denmark

5 C.N.R. Institute of Neuroscience, Padova, Italy

6 Wellcome Centre for Human Genetics, University of Oxford, Oxford, UK

7 Department of Genetic Medicine and Development, University of Geneva Medical School, Geneva, Switzerland
8 Biosciences Institute, Faculty of Medical Sciences, Newcastle University, Newcastle, UK.
9 Research Unit of Molecular Epidemiology, Helmholtz Zentrum München, German Research Center for Environmental Health, Neuherberg, Bavaria, Germany
10 Institute of Epidemiology, Helmholtz Zentrum München, German Research Center for Environmental Health, Neuherberg, Bavaria, Germany
11 Chair of Food Chemistry and Molecular and Sensory Science, Technical University of Munich, Freising, Germany
12 Metabolomics and Proteomics Core, Helmholtz Zentrum Muenchen, German Research Center for Environmental Health, Neuherberg, Germany
13 Affinity Proteomics, Science for Life Laboratory, School of Engineering Sciences in Chemistry, Biotechnology and Health, KTH Royal Institute of Technology, Solna, Sweden
14 Research and Development Global Development, Translational Medicine and Clinical Pharmacology, Sanofi-Aventis Deutschland, Frankfurt, Germany
15 Novo Nordisk Foundation Center for Basic Metabolic Research, Faculty of Health and Medical Sciences, University of Copenhagen, Copenhagen, Denmark
16 University of Exeter Medical School, Exeter, UK
17 The Immunoassay Biomarker Core Laboratory, School of Medicine, University of Dundee, Dundee, UK
18 Research Centre for Optimal Health, Department of Life Sciences, University of Westminster, London, UK
19 Section for Nutrition Research, Faculty of Medicine, Hammersmith Campus, Imperial College London, London, UK
20 Department of Radiology, Copenhagen University Hospital Herlev-Gentofte, Herlev, Denmark
21 Department of Epidemiology and Data Science, Amsterdam UMC, Vrije Universiteit Amsterdam, Amsterdam, the Netherlands
22 Department of General Practice, Amsterdam Public Health Research Institute, Amsterdam UMC, Vrije Universiteit Amsterdam, Amsterdam, the Netherlands
23 Department of Biomedical Data Science, Section Molecular Epidemiology, Leiden University Medical Center, Leiden, the Netherlands
24 Department of Cell and Chemical Biology, Leiden University Medical Center, Leiden, the Netherlands

25 Inserm, Univ Lille, CHU Lille, Lille Pasteur Institute, EGID, Lille, France
26 MRC Epidemiology Unit, University of Cambridge School of Clinical Medicine, Cambridge, UK
27 Department of Medicine, University of Eastern Finland, Kuopio, Finland
28 Institute of Cellular Medicine, Newcastle University, Newcastle, UK
29 Diabetes Research Network, Royal Victoria Infirmary, Newcastle, UK
30 Centre for Health, Law and Emerging Technologies (HeLEX), Faculty of Law, University of Oxford, Oxford, United Kingdom
31 Lund University Diabetes Centre, Department of Clinical Sciences, Lund University, Malmö, Sweden
32 Translational and Clinical Research Institute, Faculty of Medical Sciences, Newcastle University, Newcastle, UK
33 Division of Population Health & Genomics, School of Medicine, University of Dundee, Dundee, UK
34 Genetic and Molecular Epidemiology Unit, Lund University Diabetes Centre, Department of Clinical Sciences, CRC, Lund University, SUS, Malmö, Sweden
35 Eli Lilly Regional Operations, Vienna, Austria
36 Harvard T.H. Chan School of Public Health, Boston, MA, USA
37 OCDEM, Radcliffe Department of Medicine, University of Oxford, Oxford, UK
38 Institute of Experimental Genetics, Helmholtz Zentrum München, German Research Center for Environmental Health, Neuherberg, Germany
39 Department of Biochemistry, Yong Loo Lin School of Medicine, National University of Singapore, Singapore, Singapore
40 Institute of Biochemistry, Faculty of Medicine, University of Ljubljana, Ljubljana, Slovenia
41 Oxford Centre for Diabetes, Endocrinology and Metabolism, University of Oxford, Oxford, UK
42 Present address: Genentech, South San Francisco, USA

Correspondence: Simon Rasmussen (simon.rasmussen@cpr.ku.dk) and Søren Brunak (soren.brunak@cpr.ku.dk)

Supplementary Notes

Severity of drug-drug-interactions and clinical data

We investigated if any of the drug combinations had known drug-drug-interactions (DDIs). Of the 55 drug-drug pairs that were administered in our cohort we identified 52 with DDIs registered across 26 DDI databases where 12 had registered DDIs with known clinical implications ranging from 'no effect' to 'moderate effect'. We then studied if there was an association between the severity of the DDIs and the similarity of the drug-drug pairs in the multi-omics and clinical data. Here, we found that the overall omics data had small or negative associations with Spearman's Rho between -0.19 and 0.095 that were not statistically significant. The highest correlation was between reported DDIs and the clinical response with a Spearman's Rho of 0.32 (**Supplementary Figure 21)**. This could imply that the severity of the DDIs were best captured in the clinical representation whereas the omics profiles were maybe less affected by the level of severity for the DDIs with known clinical implications.

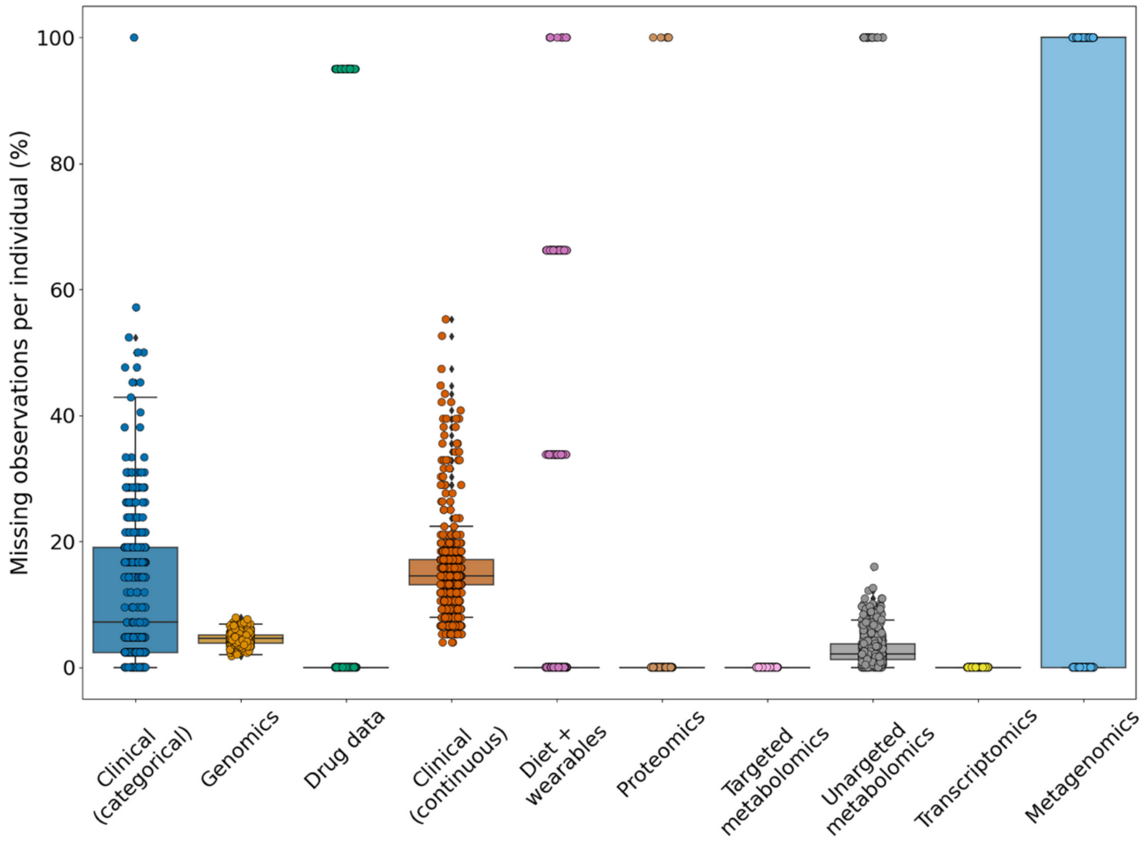

Supplementary Figure 1. Missing data across datasets. Per individual missingness (y-axis) for each of the multi-omics and clinical datasets (x-axis). The lower and upper hinges correspond to the first and third quartiles (25th and 75th percentiles). The upper and lower whiskers extend from the hinge to the highest and lowest values, respectively, but no further than 1.5 × interquartile range (IQR) from the hinge. IQR is the distance between the first and third quartiles. Data beyond the ends of whiskers are outliers and are plotted individually. Each individual datapoint is shown, however overplotting can occur, i.e. there are many more points at 100% missingness for Metagenomics than for Untargeted metabolomics. See Supplementary Table 1 for number of features in each dataset.

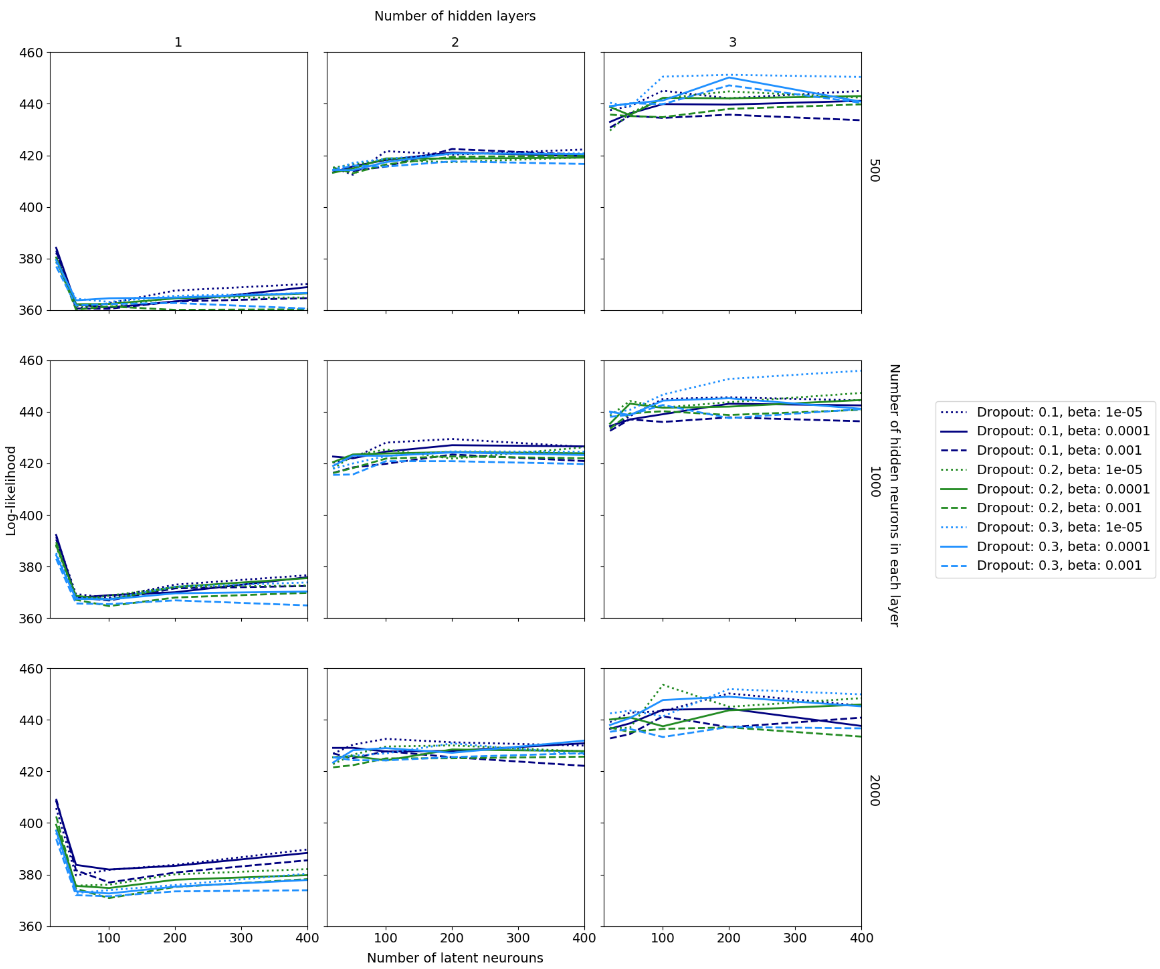

Supplementary Figure 2. Using a test set for selecting hyperparameters for the VAE. When selecting the hyperparameters for the VAE in MOVE we divided the dataset into train (90%) and test (10%) and evaluated the test log-likelihood loss (y-axis) as a function of latent neurons (x-axis), number of hidden layers (boxes from left to right), number of hidden neurons in each layer (boxes from upper to lower), dropout and weight on the Kullback-Leibler divergence (dotted and colored lines indicated in legend).

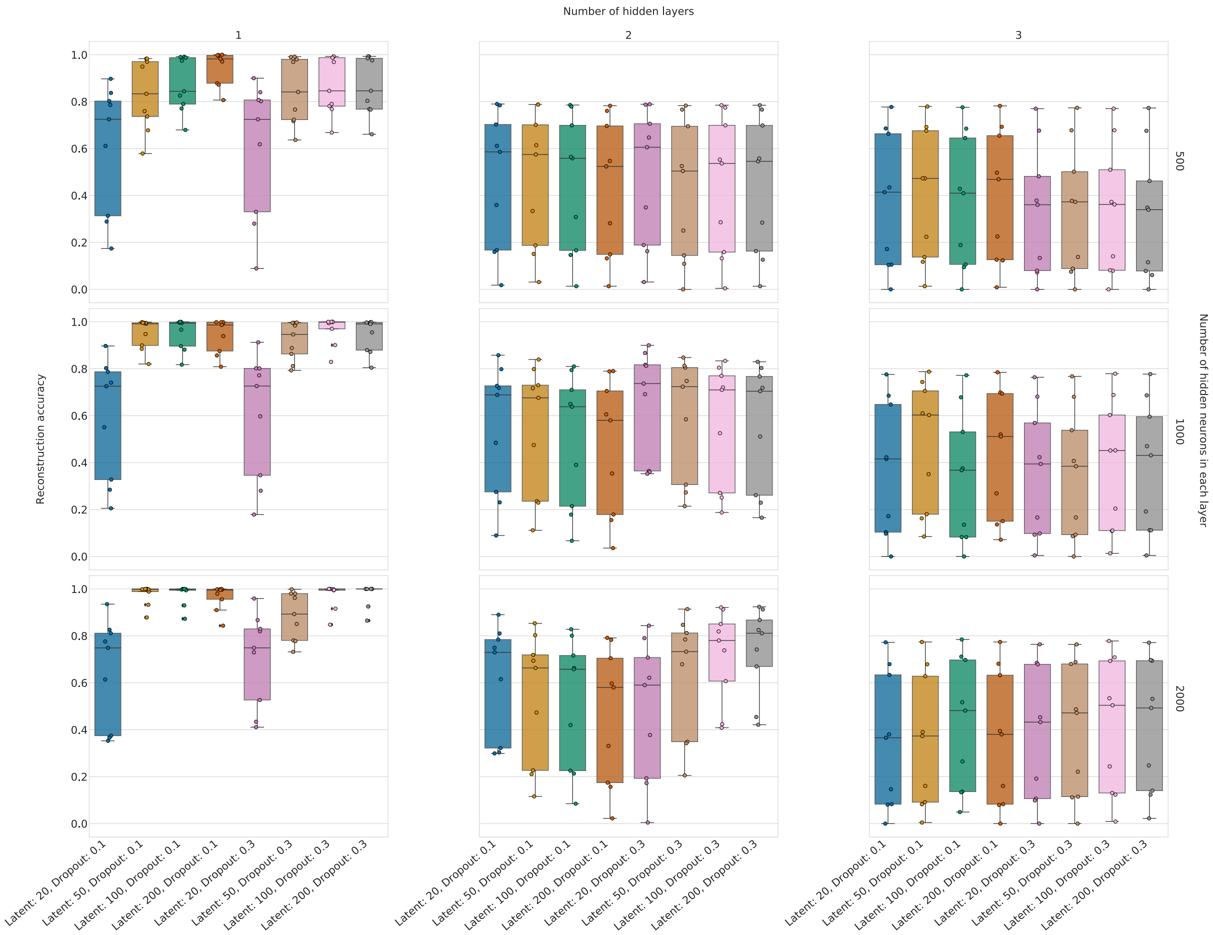

Supplementary Figure 3. Using the training set reconstruction to select hyperparameters for the VAE. In addition to assessing the test set log-likelihood we assessed the reconstructions of the training set. Here we defined an accurate reconstruction for categorical variables as the class with the highest probability corresponding to the class given by the input. For continuous variables, the accuracy was assessed by comparing the reconstructed array with the input array using cosine similarity for each individual instead of using exact matching. Sample size (n) for each of the comparisons were 10, equal to the number of data sets (omics and clinical). The lower and upper hinges correspond to the first and third quartiles (25th and 75th percentiles). The upper and lower whiskers extend from the hinge to the highest and lowest values, respectively, but no further than 1.5 × interquartile range (IQR) from the hinge. IQR is the distance between the first and third quartiles. Data beyond the ends of whiskers are outliers and are plotted individually.

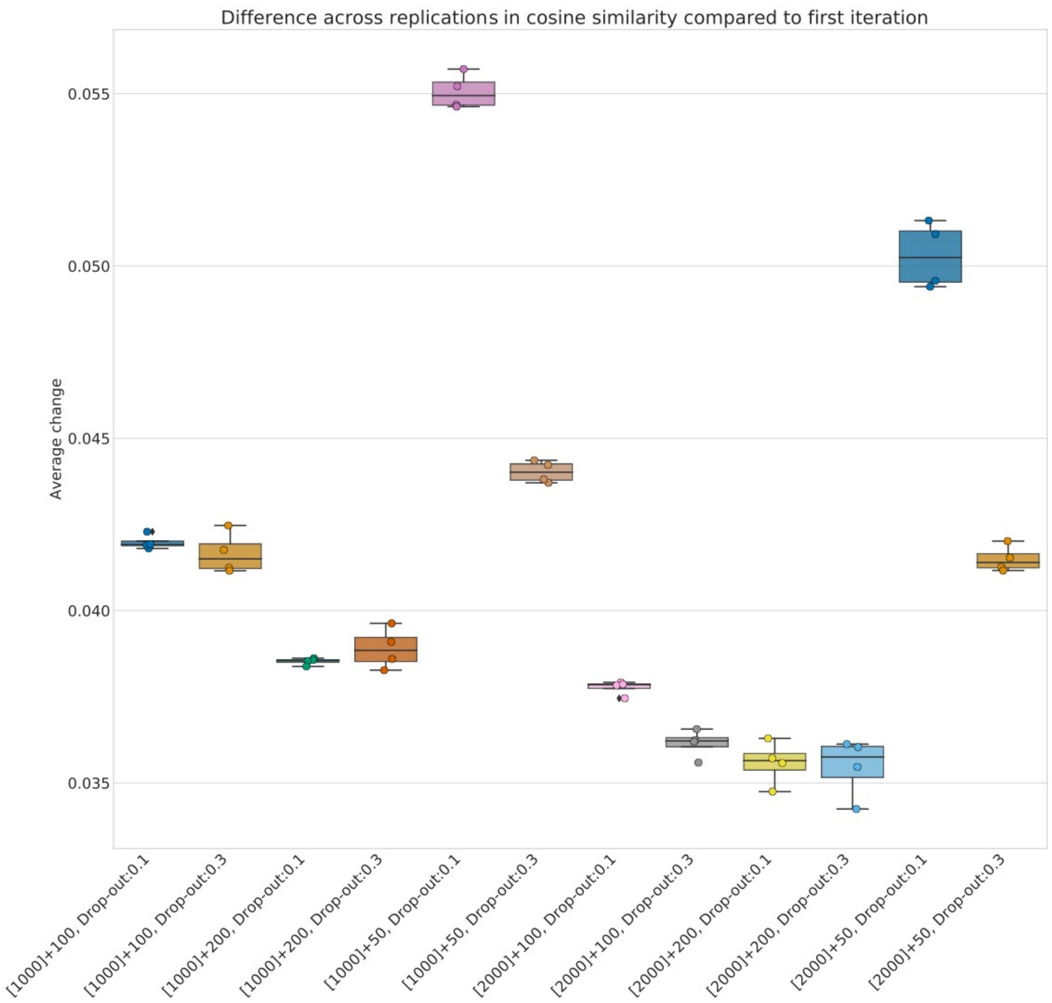

Supplementary Figure 4. Assessing reconstruction stability for selecting VAE hyperparameters. Finally, we assessed how stable the reconstructions were between runs with identical hyperparameter settings. The stability of the model was assessed by comparing cosine similarity to the same individuals when run through the first iteration and thus an average change (y-axis) of 0 indicates exactly the same reconstructions as the first iteration. For each hyperparameter combination, we ran 5 replications. The x-axis legend indicates [no. hidden neurons]+no. latent neurons, fraction dropout. The final hyperparameters were: one hidden layer of 2,000 neurons, a latent space of at least 100, and dropout of 0.1. Sample size (n) for each of the comparisons were 10, equal to the number of data sets (omics and clinical). The lower and upper hinges correspond to the first and third quartiles (25th and 75th percentiles). The upper and lower whiskers extend from the hinge to the highest and lowest values, respectively, but no further than 1.5 × interquartile range (IQR) from the hinge. IQR is the distance between the first and third quartiles. Data beyond the ends of whiskers are outliers and are plotted individually.

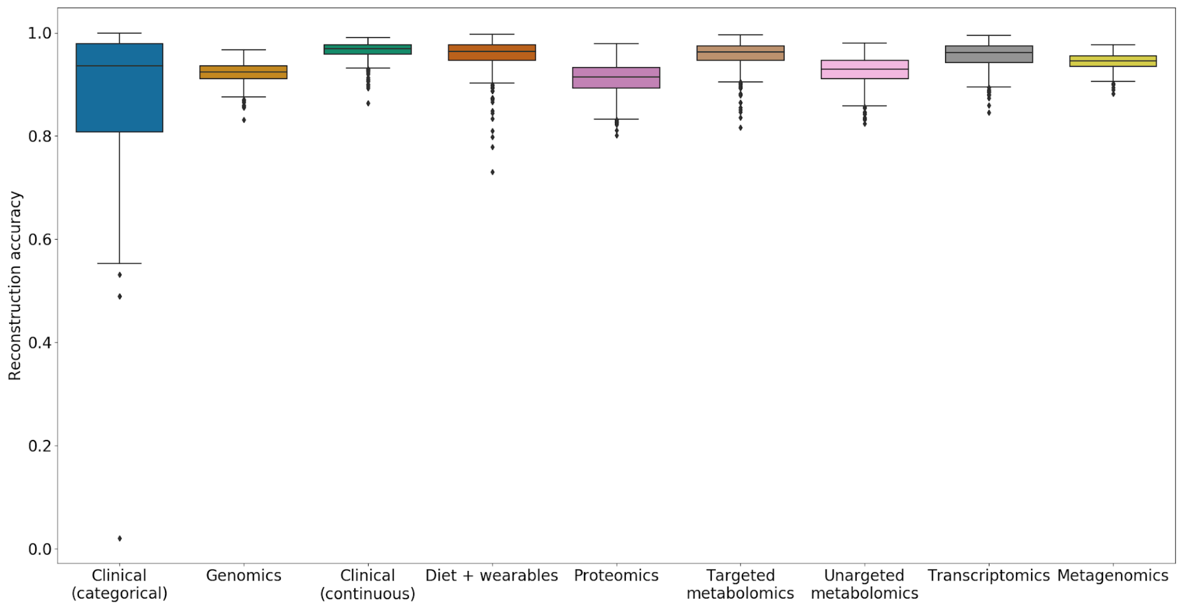

Supplementary Figure 5. Reconstruction accuracy of the selected model. Reconstruction accuracy of each dataset when using the selected hyperparameter settings on the entire dataset of the 789 individuals. As noted above, we defined an accurate reconstruction for categorical variables as the class with the highest probability corresponding to the class given by the input. For continuous variables, the accuracy was assessed by comparing the reconstructed array with the input array using cosine similarity for each individual instead of using exact matching. The lower and upper hinges correspond to the first and third quartiles (25th and 75th percentiles). The upper and lower whiskers extend from the hinge to the highest and lowest values, respectively, but no further than 1.5 × interquartile range (IQR) from the hinge. IQR is the distance between the first and third quartiles. Data beyond the ends of whiskers are outliers and are plotted individually.

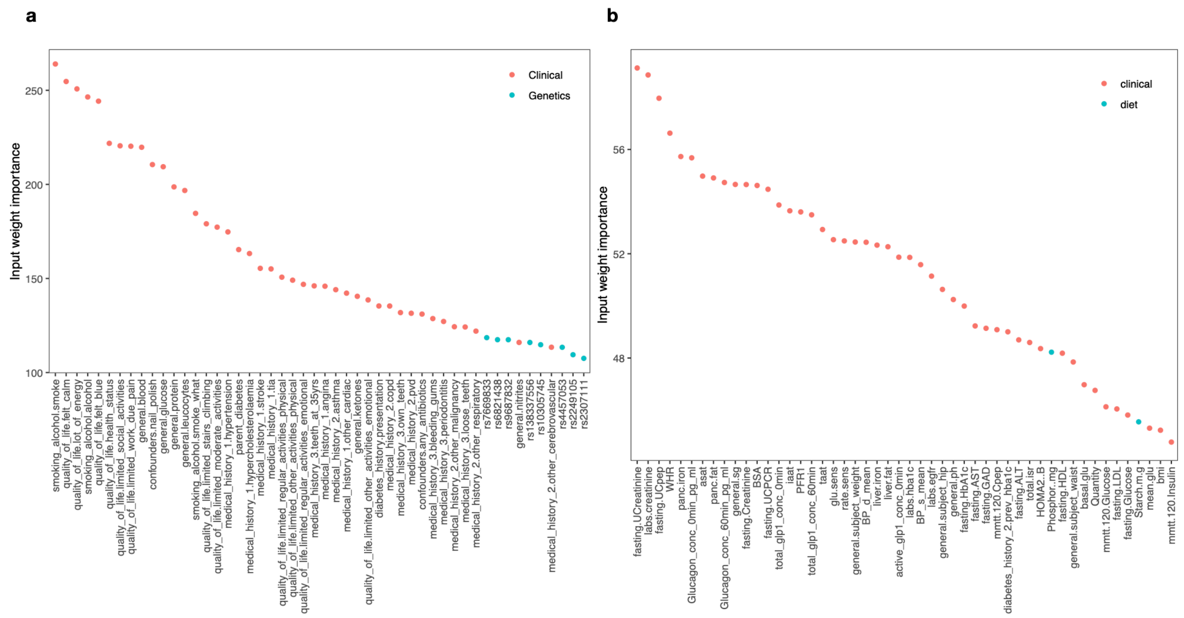

Supplementary Figure 6. MOVE first layer and latent feature importance. (a) Summed weight of the individual categorical input features to the first hidden layer in the final trained VAE. Higher values indicate that more weight is attributed to the input feature. Only the top 50 features are shown (Clinical: red, Genetics: blue). (b) As (a), but summed weight of the individual continuous input features (Clinical: red, Diet and wearables: blue). Due to the difference in encoding and number of features between the categorical and continuous features the weights cannot be directly compared between the two but should rather be used as a rank of importance.

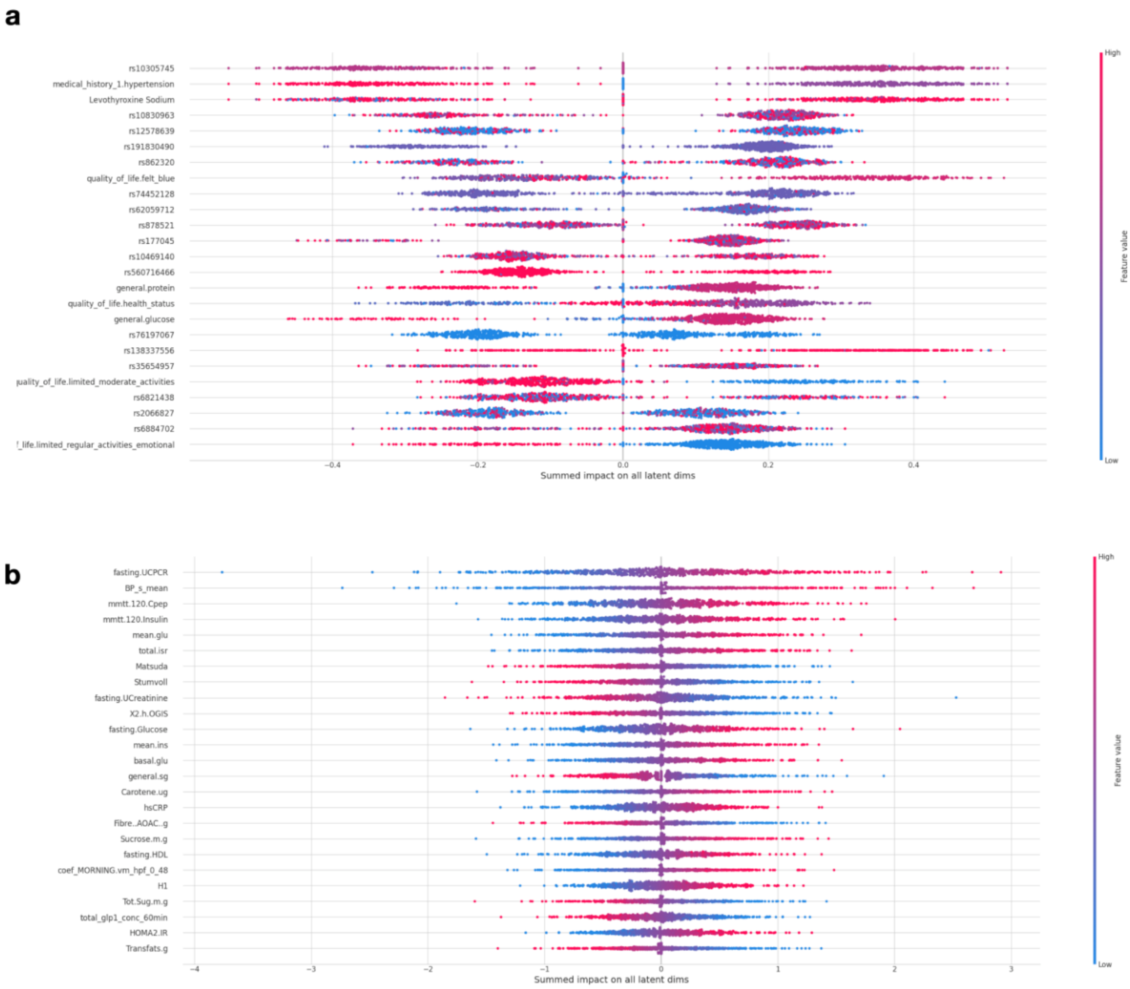

Supplementary Figure 7. SHAP analysis of the MOVE latent space. (a) SHapley Additive exPlanations (SHAP) for the discrete (a) and continuous (b) datasets showing their impact on the position of an individual in the latent space of the VAE in MOVE. The feature values are indicated as blue (low value) and red (high values). Note that the scales of the discrete features (a) are on a smaller scale than for the continuous features (b).

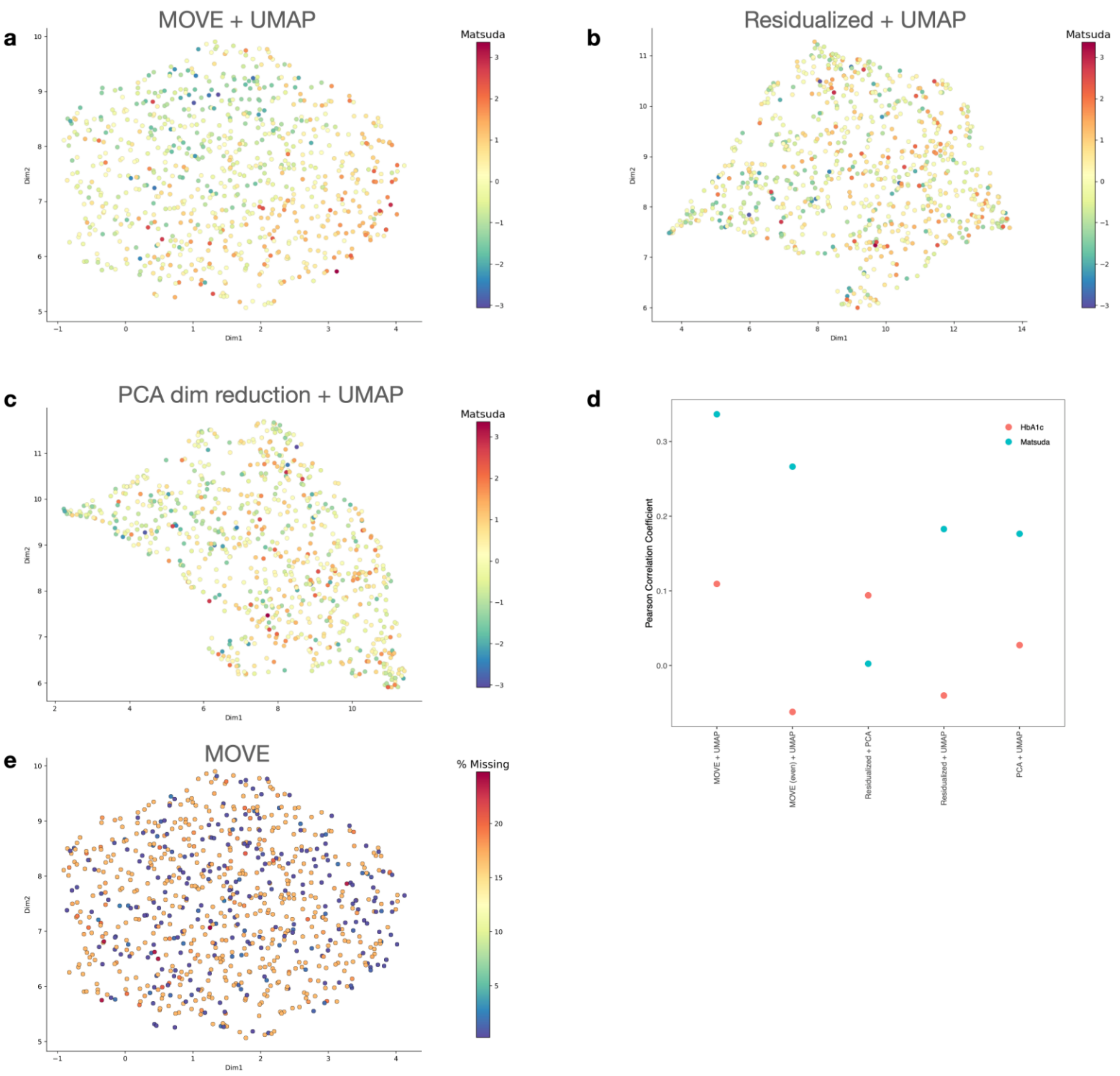

Supplementary Figure 8. MOVE identifies clinically relevant patterns. Using MOVE (a), (b) residualized data only, or (c) PCA as input to UMAP. Individuals are colored according to insulin sensitivity (Matsuda Index) from low (blue) to high (red). (d) Pearson correlation coefficient (PCC) of UMAP dimensions to HbA1c (red) and Matsuda index (blue). (e) UMAP of MOVE on residualized data where the individuals are colored according to percentage missing.

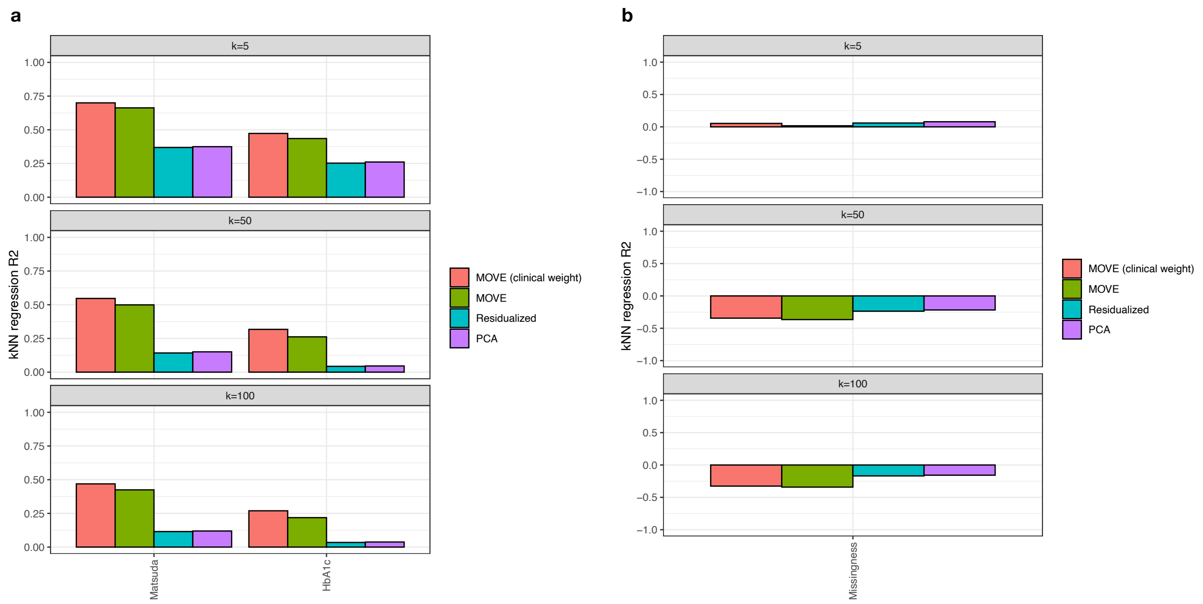

Supplementary Figure 9. kNN regression shows that MOVE identifies clinically relevant patterns. (a) kNN regression (k=5, k=50 and k=100) based on the latent representation predicting Matsuda Index and HbA1c values from the nearest neighbors indicated by *k*. R^2 (y-axis) indicates that MOVE (red and green) places the individuals with similar Matsuda Index and HbA1c values close in latent space (high R^2) in comparison to using input data (Residualized, blue) or using PCA for dimensionality reduction (purple). This can be seen for all values of *k* indicating that this is the case for both local (small *k*) and global (large *k*) area of the latent space. (b) Similar to (a) but showing the influence of missingness on local to global structure of the latent space. Missingness has very low impact on the local structure of the latent space ($R^2 < 0.06$ for MOVE).

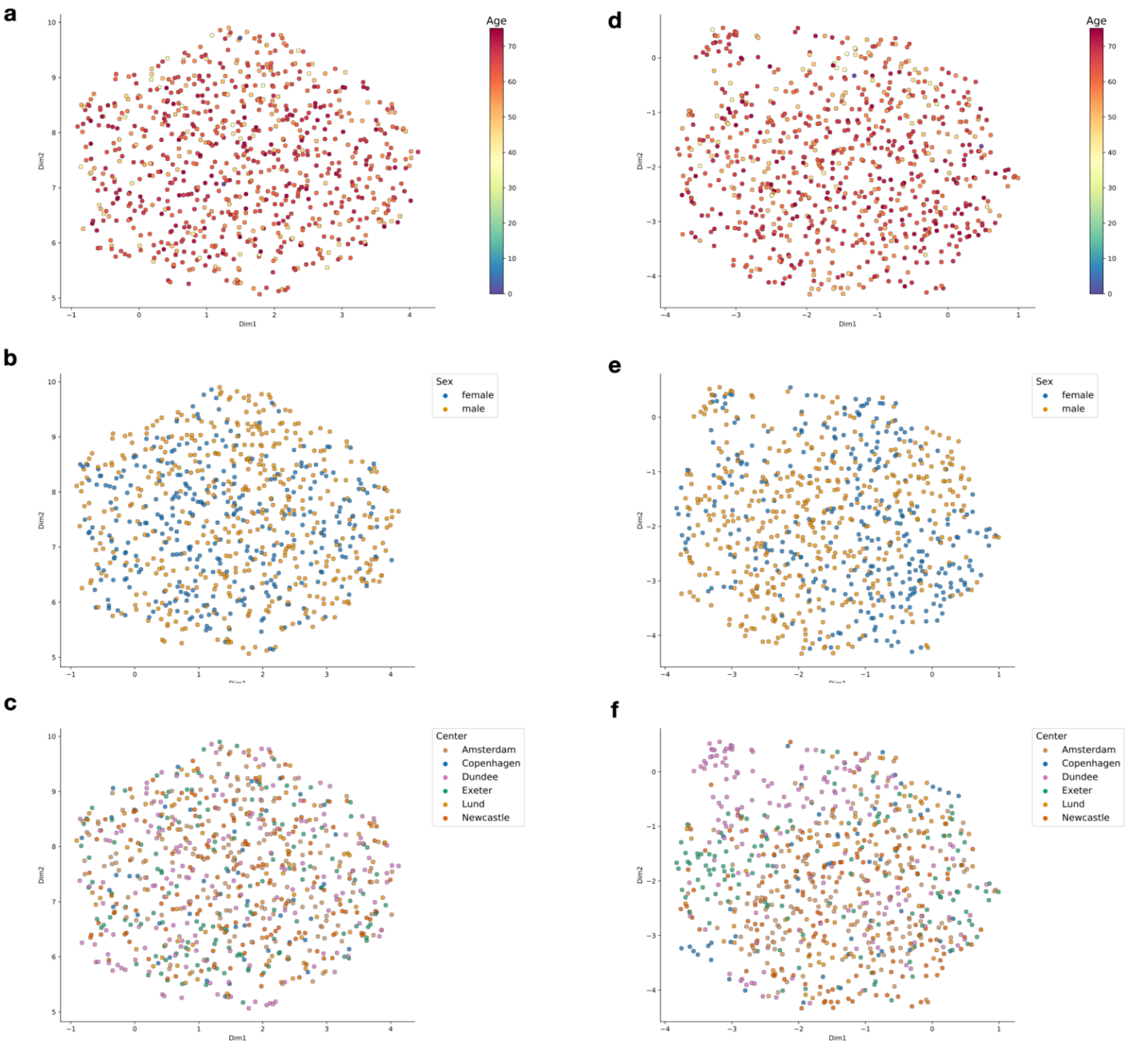

Supplementary Figure 10. MOVE is resistant to confounding effects in the data. UMAP of latent representation when applying MOVE to residualized data where the individuals are colored according to (a) age, (b) sex, and (c) center of recruitment. This can be compared to the UMAP of MOVE latent space on non-residualized data which is colored according to (d) age, (e) sex, and (f) center of recruitment.

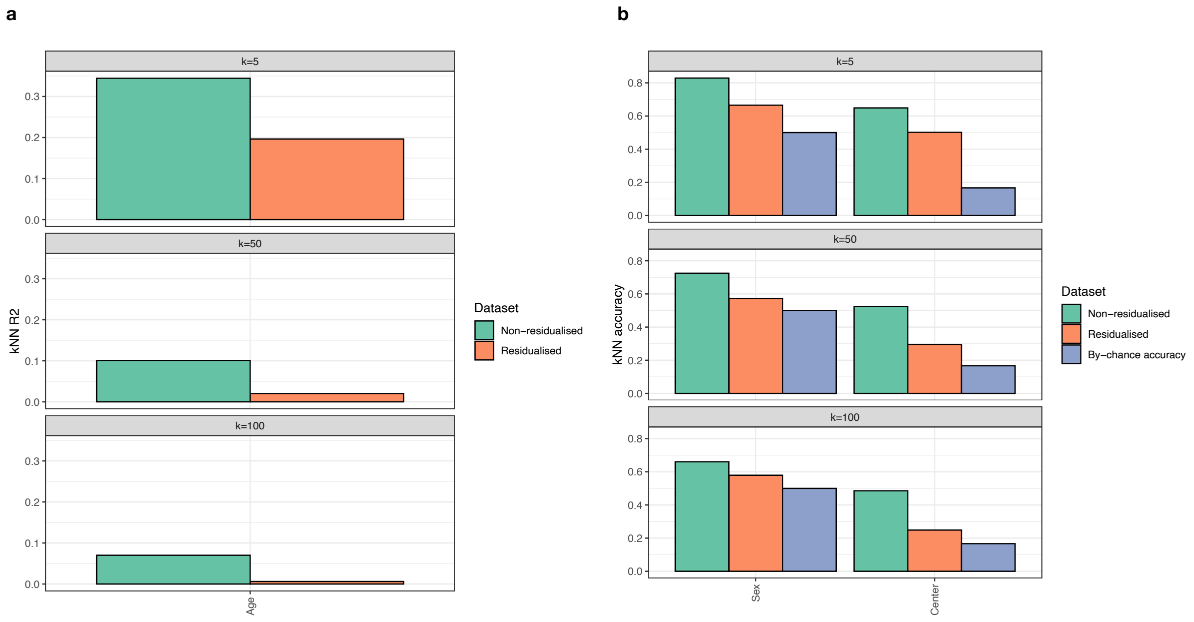

Supplementary Figure 11. kNN regression and classification shows that MOVE is resistant to confounding effects in the data. kNN regression for Age (a) and kNN classification for Sex and Center (b) using the latent representation by MOVE. Small *k* shows local structure whereas large *k* indicates a more global structure of the latent space. The plots show that residualizing (orange) reduces local and global structure of Age, Sex and Center compared to non-residualized data (green). For the residualized data there are little effect on the global structure (k=100) of Age ($R^2 < 0.01$), a small effect of Sex ($R^2 = 0.58$, by-chance accuracy 0.50 (purple)) and a small effect of recruitment center ($R^2 = 0.25$, by-chance accuracy 0.17 (purple)).

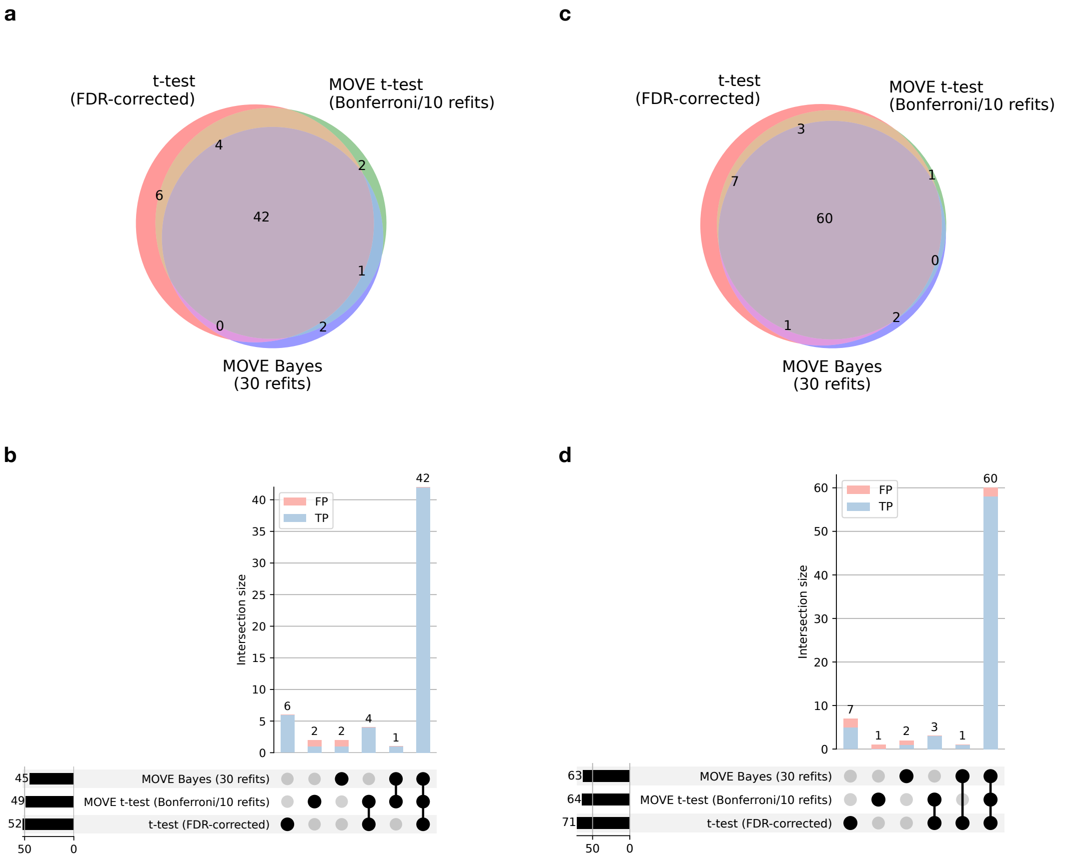

Supplementary Figure 12. Overlap between methods in the two randomized data sets. Analysis of the two randomized (shuffled) data sets using T-test on residualized data compared to MOVE T-test and MOVE Bayes. We added 100 drug-omics effects sampled from N(0,1) to each of the shuffled data sets and therefore do not expect all to be significant in the statistical tests because some effects will be close to 0. Significance threshold was set at ground truth FDR 0.05. (a) Venn diagram of overlap in TP between the methods on shuffled data set 1. (b) Upset plot showing overlap in TP and FP for the methods on shuffled data set 1. (c) As a, but for shuffled data set 2. (d) As b, but for shuffled data set 2. TP: True positive, FP: False positive.

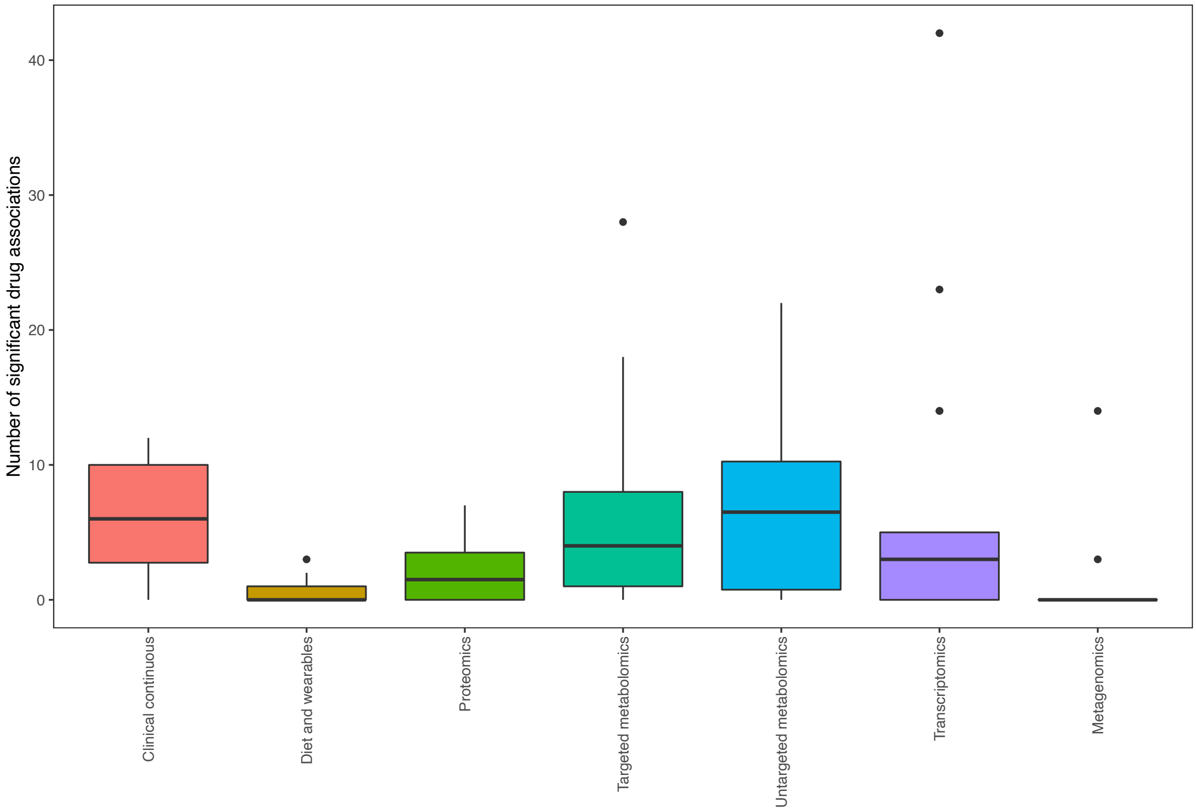

Supplementary Figure 13. Absolute number of significant hits. The number of features in the multi-omics datasets that were found by MOVE to be significantly associated with a drug (n=20). The lower and upper hinges correspond to the first and third quartiles (25th and 75th percentiles). The upper and lower whiskers extend from the hinge to the highest and lowest values, respectively, but no further than 1.5 × interquartile range (IQR) from the hinge. IQR is the distance between the first and third quartiles. Data beyond the ends of whiskers are outliers and are plotted individually.

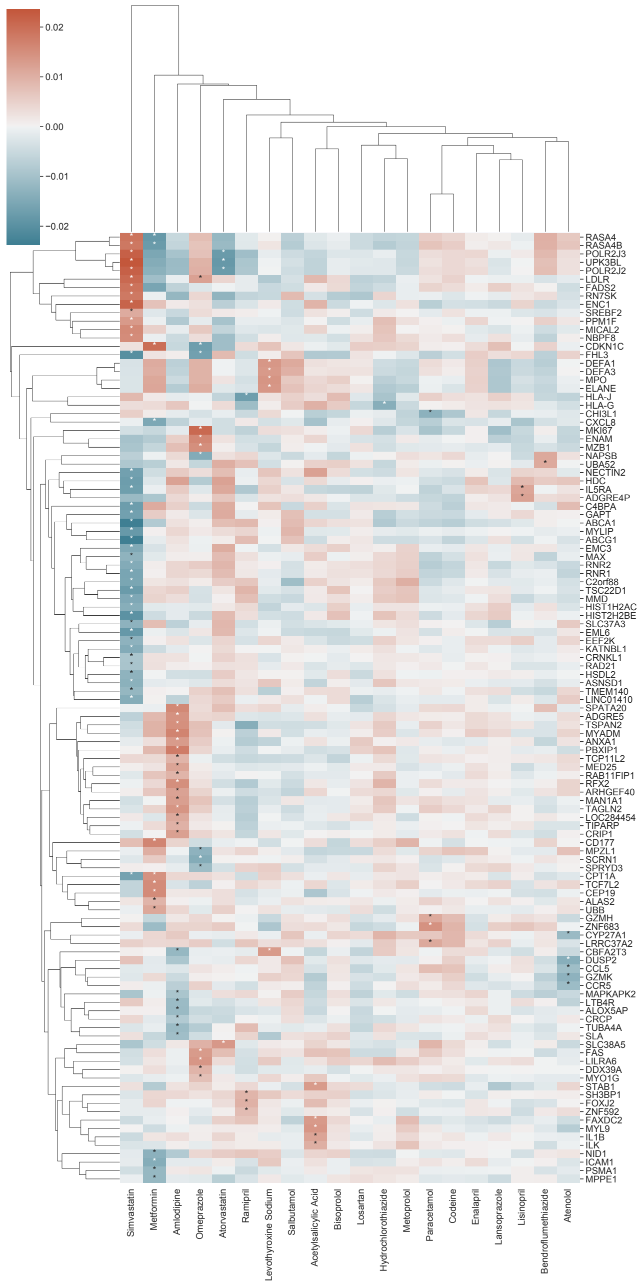

Supplementary Figure 14. Association of drugs to transcriptomics data. All transcriptomics features (y-axis) with at least one significant association to a drug (x-axis). The names of the features are given on the y-axis and can also be found in **Supplementary Data 4**. Significant drug and transcriptomics associations are indicated with an asterisk. Z-scaled effect size is indicated from negative (blue) to positive (red).

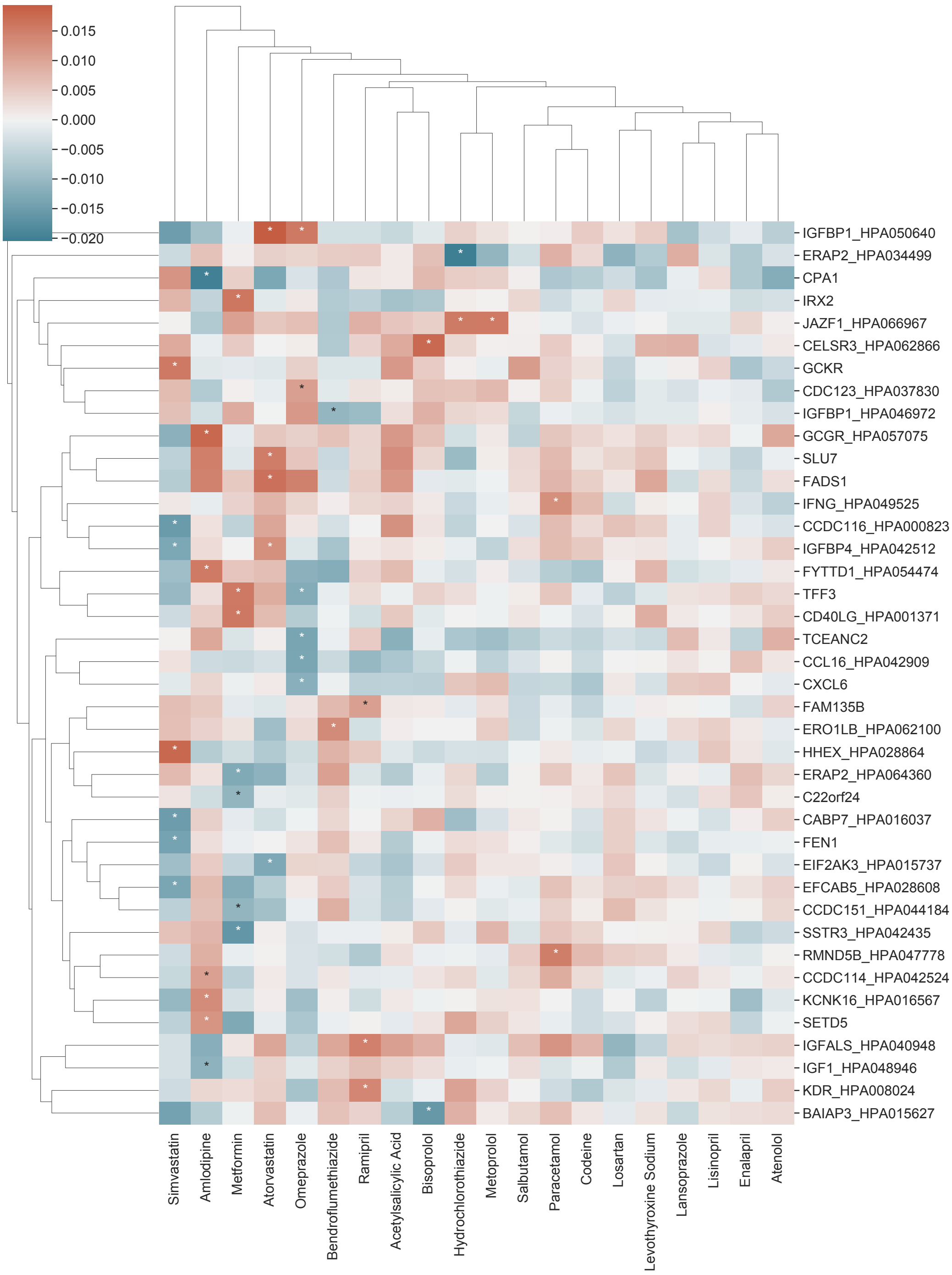

Supplementary Figure 15. Association of drugs to proteomics data. All proteomics features (y-axis) with at least one significant association to a drug (x-axis). The names of the features are given on the y-axis and can also be found in **Supplementary Data 4**. Significant drug and proteomics associations are indicated with an asterisk. Z-scaled effect size is indicated from negative (blue) to positive (red).

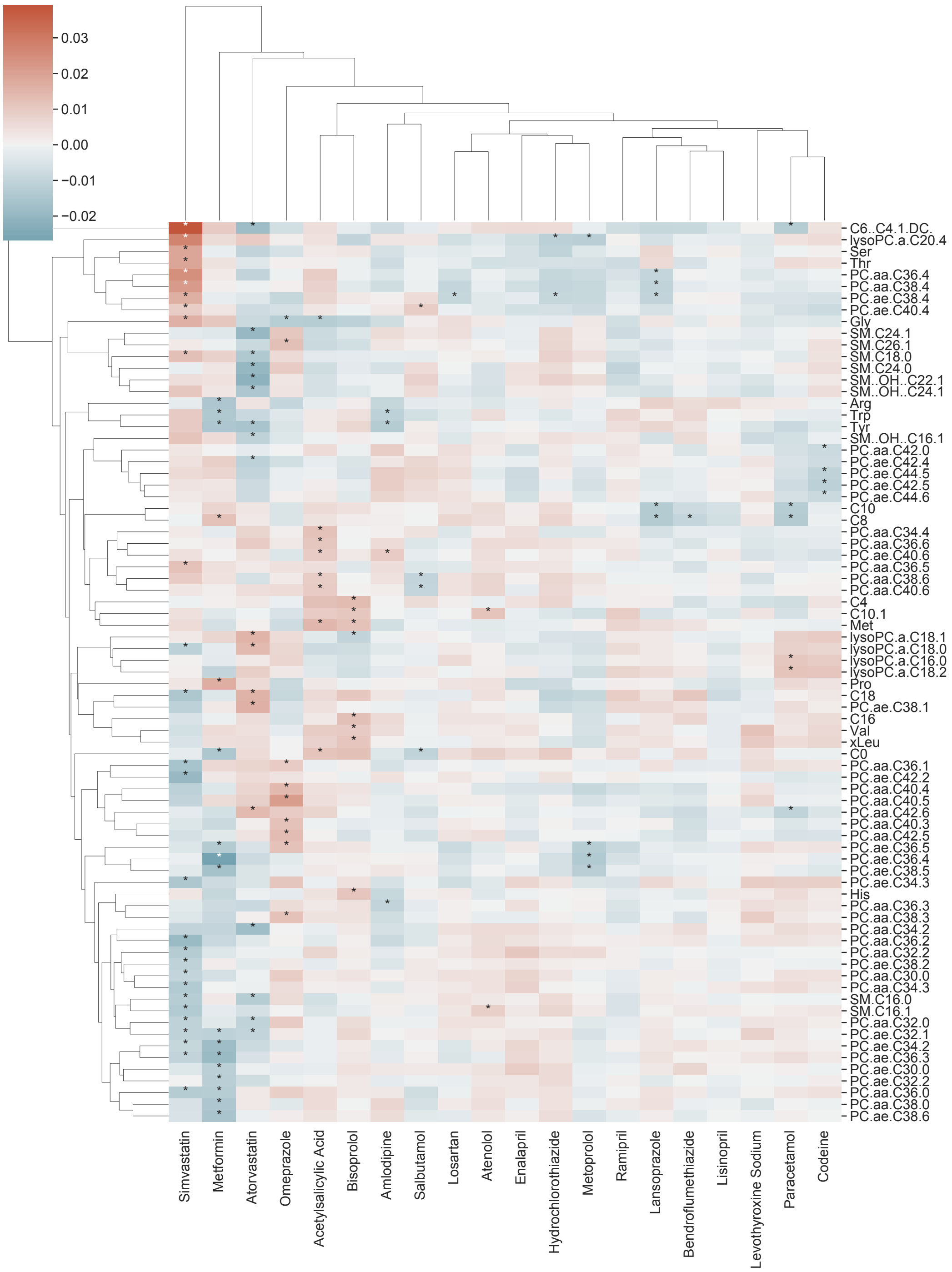

Supplementary Figure 16. Associations of drugs to targeted metabolomics data. All targeted metabolomics features (y-axis) with at least one significant association to a drug (x-axis) are shown. The names of the metabolomics features are given on the y-axis and can also be found in **Supplementary Data 4**. Significant drug and metabolomics associations are indicated with an asterisk. Z-scaled effect size is shown from negative (blue) to positive (red).

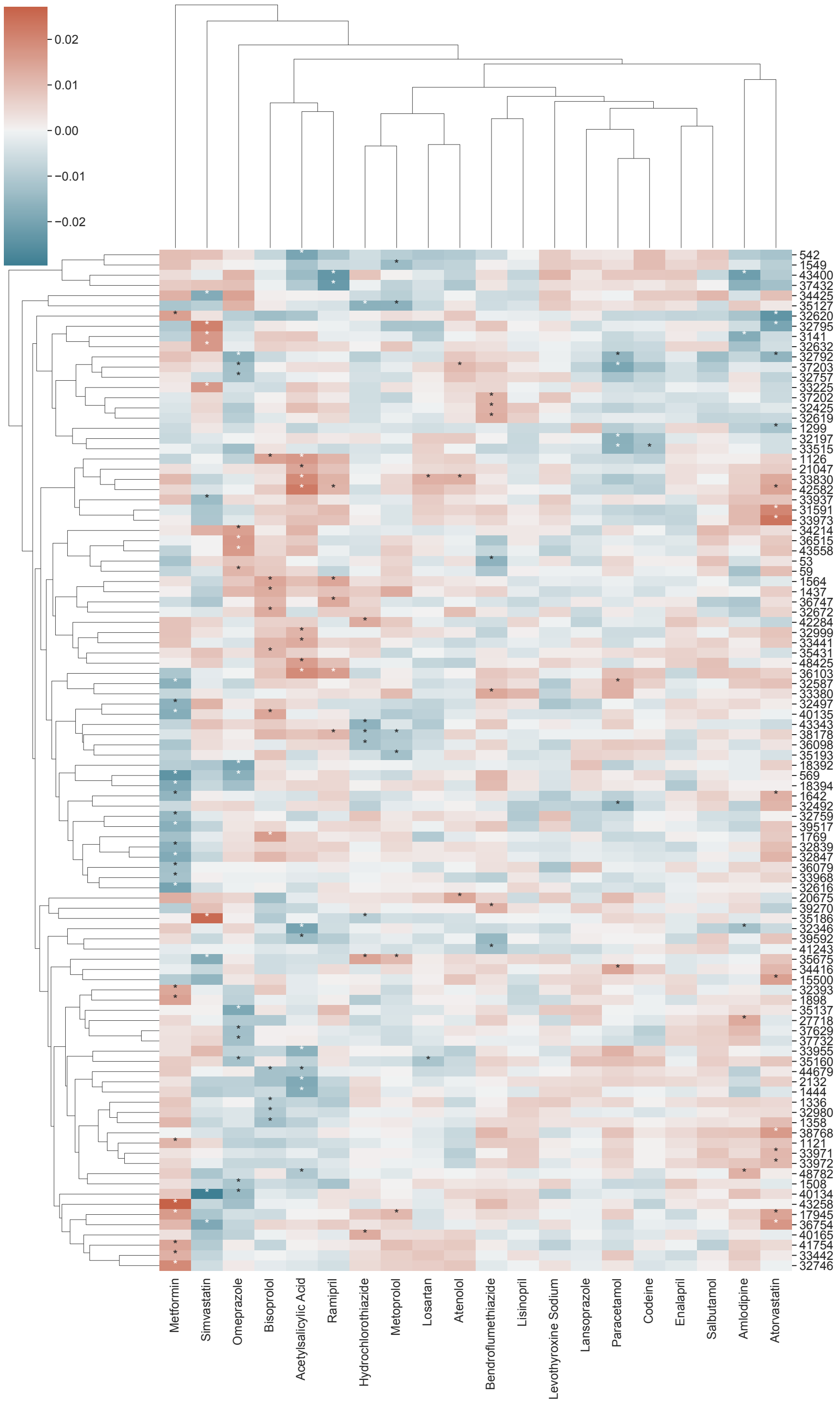

Supplementary Figure 17. Associations of drugs to untargeted metabolomics data. All untargeted metabolomics features (y-axis) with at least one significant association to a drug (x-axis) are shown. The names of the metabolomics features are not indicated but the data can be found in **Supplementary Data 4**. Significant drug and metabolomics associations are indicated with an asterisk. Z-scaled effect size is shown from negative (blue) to positive (red).

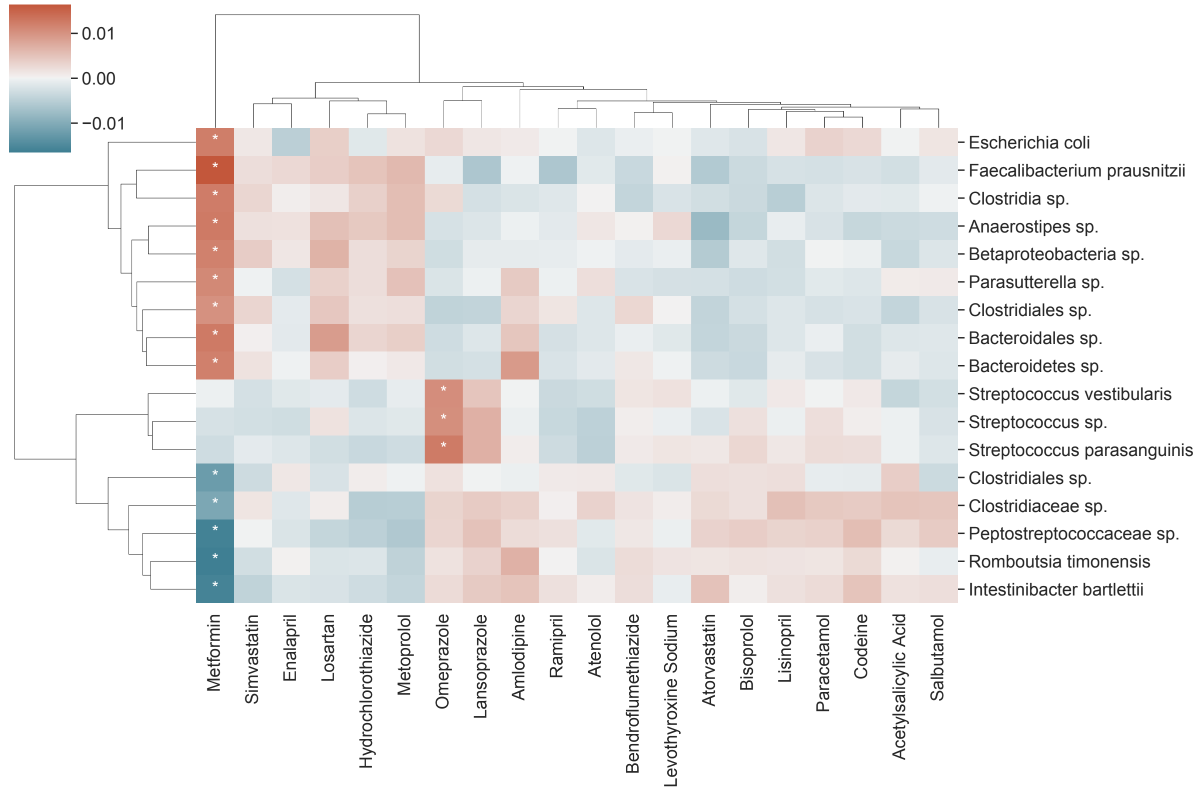

Supplementary Figure 18. Associations of drugs to metagenomics data. All metagenomics species (y-axis) with at least one significant association to a drug (x-axis) is shown. The name of the metagenomics species is indicated on the y-axis and can additionally be found in **Supplementary Data 4**. Significant drug and metagenomics associations are indicated with an asterisk. Z-scaled effect size is shown from negative (blue) to positive (red).

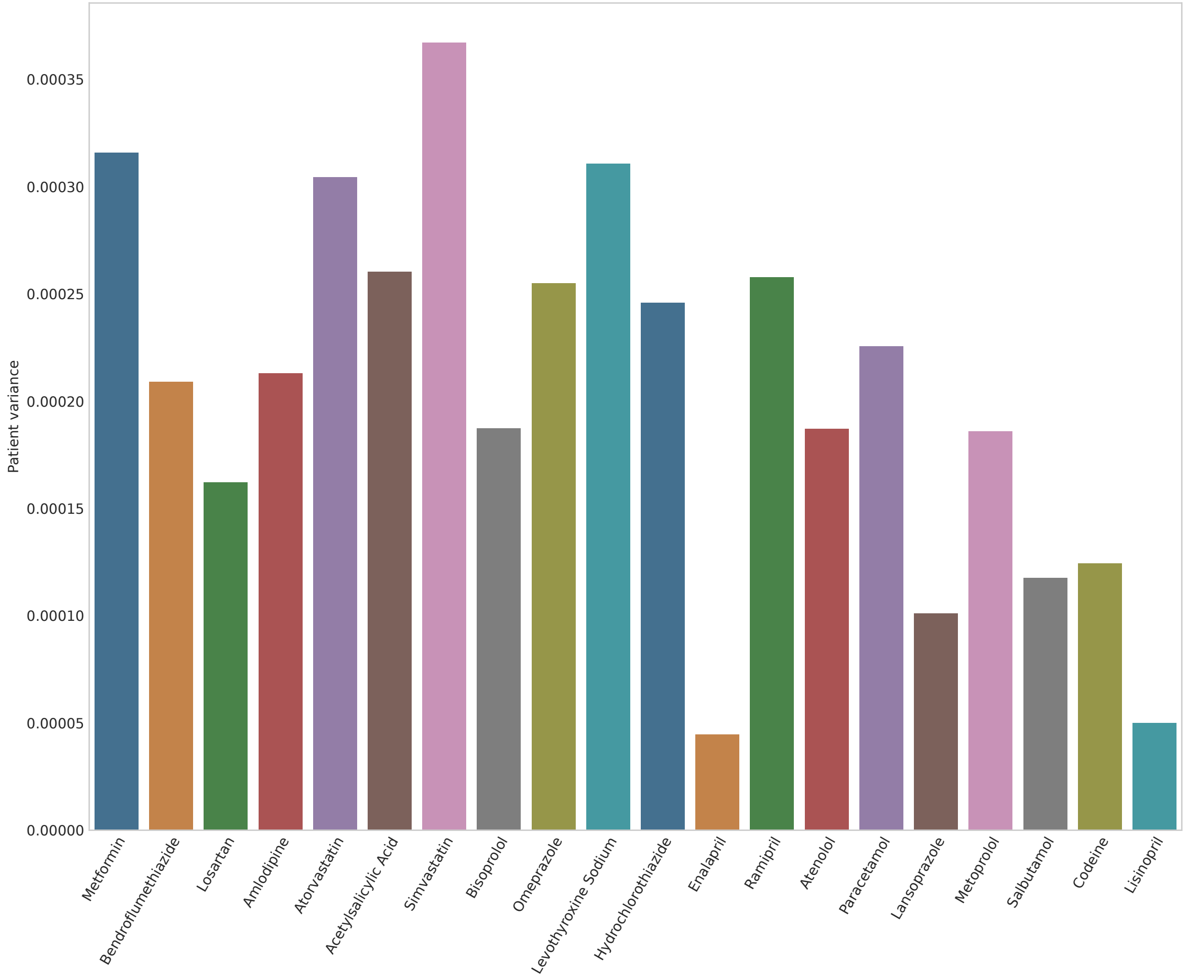

Supplementary Figure 19. Inter person variation of significant drug multi-omics associations. The variance in effect size predicted from individuals across the multi-omics datasets.

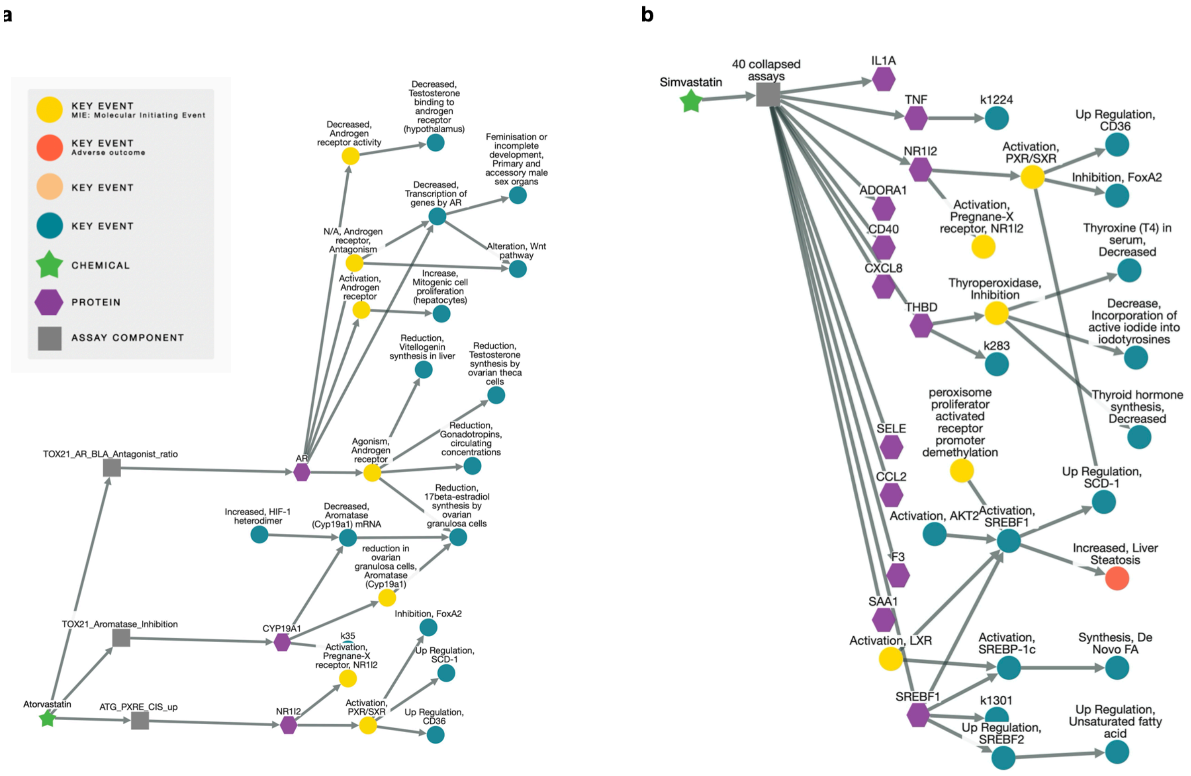

Supplementary Figure 20. Adverse outcome pathways. Adverse outcome pathway for atorvastatin (a) and simvastatin (b). Legend is indicated in (a).

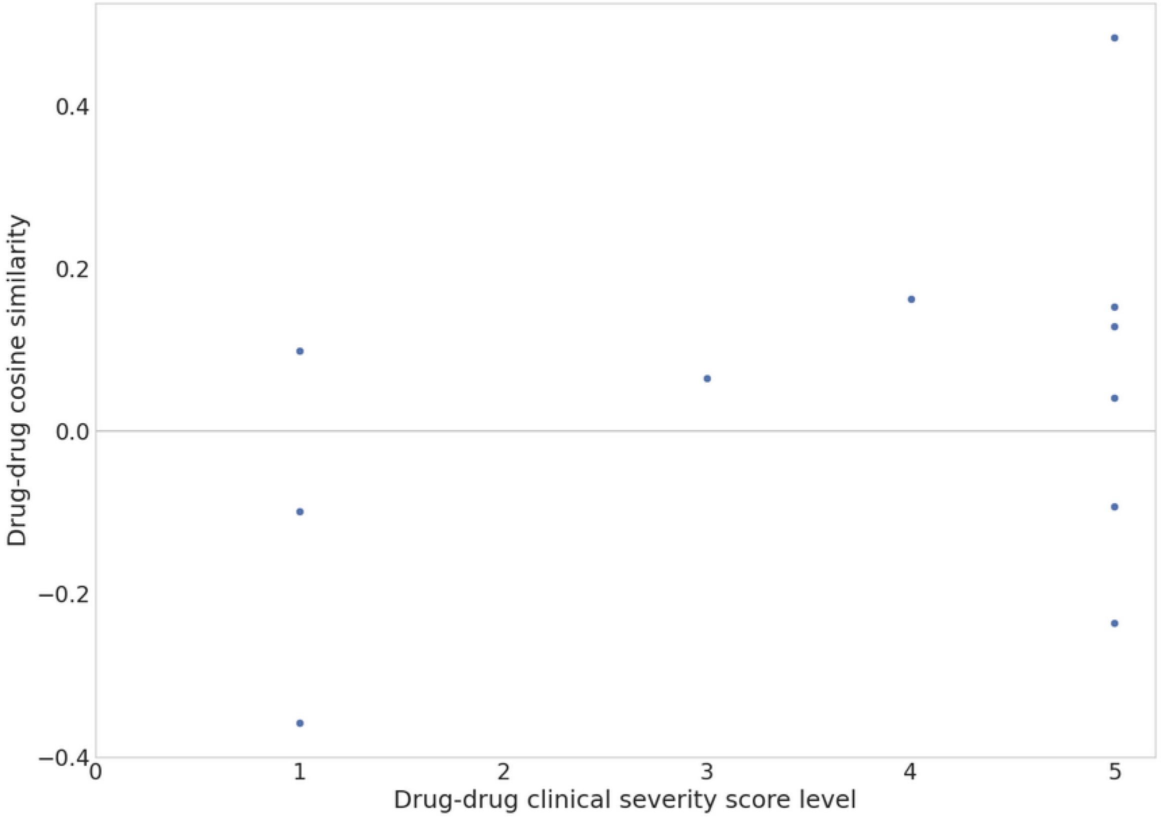

Supplementary Figure 21. Correlation between drug-drug omics similarity and known drug-drug interactions. Cosine similarity in the clinical changes between every drug-drug combination with a known clinical severity score plotted against the level of clinical severity. The score ranges from 1-5 translated to "no effect" (1), "undetermined" (2), "possible effect" (3), "minor effect" (4) or "moderate effect" (5). Two drug-drug interactions with undetermined effects (2 on the x-axis) were removed for the figure and correlation analyses.

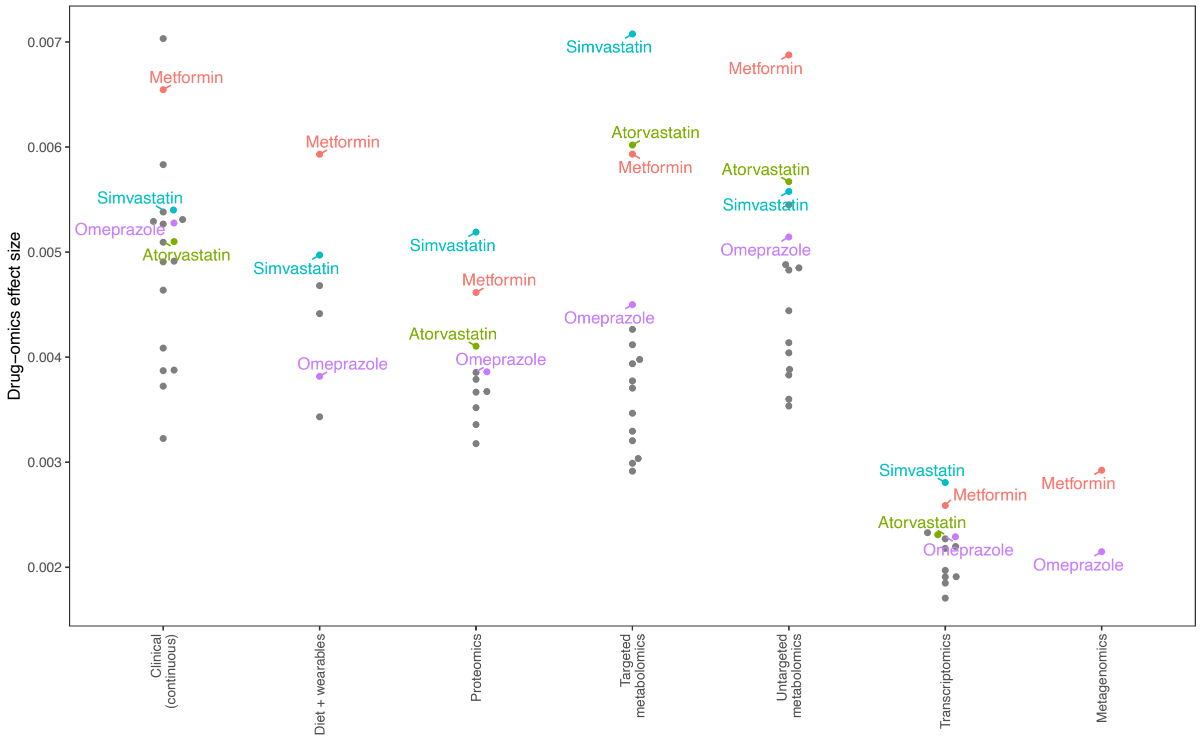

Supplementary Figure 22. Effect sizes of drug and multi-omics associations stratified per omics dataset. The z-scaled effect size (y-axis) of the significant drug and multi-omics features was stratified by the omics dataset (x-axis). As Figure 3d, but only including significant drug-omics associations. Drugs with no significant omics association for a particular data set have been omitted.

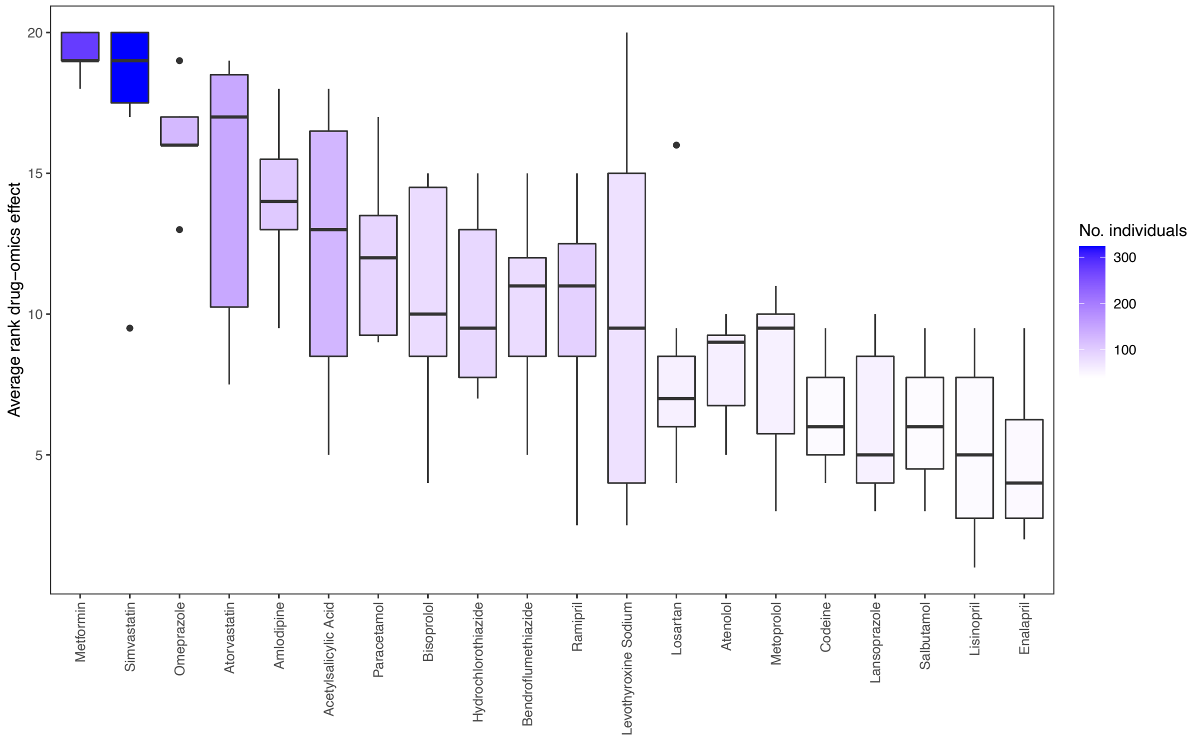

Supplementary Figure 23. Distribution of multi-omics ranks for the different drugs when only considering significant hits. The ranks are determined as a number between 1-20 (drugs) based on the average effect size shown in Supplementary Figure 18. The boxes are colored according to number of individuals taking a particular drug from 0 (white) to 391 (purple). There was a correlation between number of individuals taking a drug and the average rank (PCC=0.86). The lower and upper hinges correspond to the first and third quartiles (25th and 75th percentiles). The upper and lower whiskers extend from the hinge to the highest and lowest values, respectively, but no further than 1.5 × interquartile range (IQR) from the hinge. IQR is the distance between the first and third quartiles. Data beyond the ends of whiskers are outliers and are plotted individually.

Dataset	Clinical (continuous)	Diet and wearables	Prote-omics	Targeted metabol-omics	Untargeted metabol-omics	Transcript -omics	Metagen -omics
Number of features	76	74	373	119	238	6,018	1,463

Dataset	Clinical (categorical)	Genomics	Drug data
Number of features	42	393	20

Supplementary Table 1. Size of included datasets. Number of features included in the input dataset. The upper table shows continuous features whereas the lower table has categorical features.

Drug	Metformin	Bendroflumethiazide	Losartan	Amlodipine	Atorvastatin	Acetylsalicylic Acid	Simvastatin	Bisoprolol	Omeprazole	Levothyroxine Sodium	Hydrochlorothiazide	Enalapril	Ramipril	Atenolol	Paracetamol	Lansoprazole	Metoprolol	Salbutamol	Codeine	Lisinopril
Metformin	277																			
Bendroflumethiazide	20	82																		
Losartan	26	4	58																	
Amlodipine	36	17	15	105																
Atorvastatin	46	15	8	42	146															
Acetylsalicylic Acid	43	17	7	27	42	129														
Simvastatin	123	37	23	33	1	58	323													
Bisoprolol	23	6	3	10	27	43	31	82												
Omeprazole	45	10	10	17	29	30	48	20	125											
Levothyroxine Sodium	31	9	5	9	21	13	29	8	17	76										
Hydrochlorothiazide	43	0	15	11	10	14	38	5	10	11	87									
Enalapril	20	5	1	2	9	6	20	5	5	9	14	47								
Ramipril	32	20	0	17	35	40	39	27	14	7	4	0	96							
Atenolol	19	12	7	9	18	19	23	0	7	4	5	7	14	59						
Paracetamol	31	18	5	19	26	16	35	14	26	15	7	2	14	5	90					
Lansoprazole	21	9	1	13	16	8	26	9	1	3	3	0	8	4	19	57				
Metoprolol	37	3	9	6	10	19	22	0	8	4	28	6	7	0	5	1	57			
Salbutamol	17	2	4	6	14	9	19	5	11	4	1	1	9	1	12	6	0	45		
Codeine	13	6	1	7	16	5	20	4	13	9	3	3	5	3	38	9	1	6	46	
Lisinopril	12	10	1	9	12	11	21	5	6	6	6	0	0	2	10	9	4	3	6	46

Supplementary Table 2. Number of individuals administered a certain drug or drug combination. Number of individuals taking each of the drug combinations included in the study. The diagonal represents the number of individuals that takes the drug. Individuals taking a combination within the same therapeutic ATC group was not included in the analysis of those drugs.

			Shuffle 1				Shuffle 2			
**Method**	**Multiple correction**	**Refits**	**TP**	**FP**	**FDR (truth)**	**AUC**	**TP**	**FP**	**FDR (truth)**	**AUC**
T-test	Bonferroni	NA	49	0	0.00	0.93	62	4	0.06	0.91
T-test	FDR BH 0.05	NA	56	6	0.10	0.92	67	12	0.15	0.91
**T-test***	FDR BH 0.01	NA	52	0	0.00	0.92	67	4	0.06	0.91
T-test	FDR BH 0.001	NA	49	0	0.00	0.92	62	4	0.06	0.91
T-test	FDR BH 0.0001	NA	43	0	0.00	0.92	59	3	0.05	0.91
MOVE T-test	MS-Bonferroni	1	44	10	0.19	0.86	60	11	0.15	0.86
MOVE T-test	MS-Bonferroni	5	47	2	0.04	0.89	60	4	0.06	0.88
**MOVE T-test***	MS-Bonferroni	10	48	1	0.02	0.90	61	3	0.05	0.88
MOVE T-test	MS-Bonferroni	15	46	1	0.02	0.90	61	2	0.03	0.89
MOVE T-test	MS-Bonferroni	20	47	2	0.04	0.91	62	2	0.03	0.89
MOVE T-test	MS-Bonferroni	30	48	1	0.02	0.91	61	2	0.03	0.90
MOVE T-test	MS-Bonferroni	35	48	0	0.00	0.91	60	2	0.03	0.89
MOVE T-test	MS-Bonferroni	40	48	1	0.02	0.91	61	2	0.03	0.89
MOVE T-test	MS-Bonferroni	50	48	0	0.00	0.91	61	2	0.03	0.88
MOVE Bayes	FDR Bayes 0.05	1	0	0	NA	0.86	0	0	NA	0.85
MOVE Bayes	FDR Bayes 0.05	5	5	0	0.00	0.92	2	0	0.00	0.89
MOVE Bayes	FDR Bayes 0.05	10	16	0	0.00	0.92	17	1	0.06	0.88
MOVE Bayes	FDR Bayes 0.05	15	26	0	0.00	0.93	35	1	0.03	0.92
MOVE Bayes	FDR Bayes 0.05	20	34	0	0.00	0.93	45	1	0.02	0.92
**MOVE Bayes***	FDR Bayes 0.05	30	44	1	0.02	0.92	60	3	0.05	0.91
MOVE Bayes	FDR Bayes 0.05	35	46	3	0.06	0.93	60	8	0.12	0.92
MOVE Bayes	FDR Bayes 0.05	40	49	5	0.09	0.93	62	11	0.15	0.91
MOVE Bayes	FDR Bayes 0.05	50	54	10	0.16	0.93	65	19	0.23	0.91

Supplementary Table 3. Selecting number of variational refits and determining ground truth FDR. We selected the number of variational refits for the MOVE T-test and MOVE Bayes tests models based on shuffling the input data and adding 100 drug-omics effects sampled from N(0,1). We repeated the shuffling resulting in two data sets, Shuffle 1 and Shuffle 2. Here we found that the MOVE T-test approach had high TP and low FP from 10 refits with ground truth FDR of ~0.05. Note that the MOVE T-test approach is an ensemble of four different models and thus 10 refits represent 40 refits across the 4 models in total. For the MOVE Bayes test approach, we identified 30 refits as the optimal model. For comparison to a standard T-test we found Benjamini-Hochberg correcting the p-values to 0.01 was equivalent of a ground truth FDR of 0.05. Models indicated with an asterisks and bold were the ones chosen. MS-Bonferroni: Multi-stage Bonferroni as we use Bonferroni correction for

each model and enforce drug-omics associations to be significant in at least 50% of refits and in 3 of 4 models. FDR: False Discovery Rate, BH: Benjamini-Hochberg, TP: True Positive, FP: False Positive, AUC: Area Under Curve.

Drug	Clinical continuous	Diet and wearables	Prote-omics	Targeted metabolomics	Untargeted metabolomics	Transcript-omics	Metagen-omics
Acetylsalicylic Acid	10	0	0	8	16	5	0
Amlodipine	7	2	7	4	5	23	0
Atenolol	3	0	0	2	3	5	0
Atorvastatin	4	0	5	18	14	4	0
Bendroflumethi azide	2	0	2	1	7	1	0
Bisoprolol	10	0	2	8	11	0	0
Codeine	2	0	0	4	1	0	0
Enalapril	1	0	0	0	0	0	0
Hydrochlorothi azide	6	0	2	2	8	1	0
Lansoprazole	2	0	0	5	0	0	0
Levothyroxine Sodium	12	3	0	0	0	5	0
Lisinopril	0	0	0	0	0	2	0
Losartan	10	0	0	1	2	0	0
Metformin	12	2	7	17	22	14	14
Metoprolol	3	0	1	4	6	0	0
Omeprazole	10	2	6	9	15	14	3
Paracetamol	6	1	2	6	7	4	0
Ramipril	9	0	3	0	7	4	0
Salbutamol	8	0	0	4	0	0	0
Simvastatin	5	1	7	28	10	42	0
Total	122	11	44	121	134	124	17

Supplementary Table 4. Significant associations between drugs and omics datasets from the overlap between MOVE T-test and MOVE Bayes.

**Drug**	**ATC subgroups**	**Individuals with drug**	**No drug data**	**Individuals with ATC drug**	**Individuals without ATC drug**
Acetylsalicylic Acid	B01,N02,A01	129	68	203	518
Amlodipine	C08	105	68	105	616
Atenolol	C07	59	68	198	523
Atorvastatin	C10	146	68	468	253
Bendroflumethiazide	C03	82	68	169	552
Bisoprolol	C07	82	68	198	523
Codeine	R05	46	68	46	675
Enalapril	C09	47	68	245	476
Hydrochlorothiazide	C03	87	68	169	552
Lansoprazole	A02	57	68	181	540
Levothyroxine Sodium	H03	76	68	76	645
Lisinopril	C09	46	68	245	476
Losartan	C09	58	68	245	476
Metformin	A10	277	0	277	512
Metoprolol	C07	57	68	198	523
Omeprazole	A02	125	68	181	540
Paracetamol	N02	90	68	203	518
Ramipril	C09	96	68	245	476
Salbutamol	R03	45	68	45	676
Simvastatin	C10	323	68	468	253

Supplementary Table 5. Sample sizes (n) for the association analyses of drugs vs. multi-omics features. We calculated significantly associated drug multi-omics associations using the MOVE t-test and MOVE Bayes frameworks. By comparing baseline models (no change in drug status) with models where the drug status was changed from 0 to 1, we could identify significant drug multi-omics associations. The sample size for each of the drug tests was therefore the number of individuals (789) subtracting the number of individuals administered the drug within the same ATC class and the number of individuals with unknown status for the particular drug (‘Individuals without ATC drug’).

Drug	T-test	ANOVA	MOVE T-test	MOVE Bayes	MOVE Overlap	Diff MOVE Overlap vs T-test
Acetylsalicylic Acid	1	1	297	54	39	38
Amlodipine	0	0	352	59	48	48
Atenolol	0	0	82	14	13	13
Atorvastatin	71	71	62	65	45	-26
Bendroflumethiazide	2	2	125	19	13	11
Bisoprolol	0	0	124	38	31	31
Codeine	2	2	123	10	7	5
Enalapril	0	0	23	1	1	1
Hydrochlorothiazide	0	0	214	21	19	19
Lansoprazole	3	1	60	13	7	4
Levothyroxine Sodium	0	0	187	32	20	20
Lisinopril	1	2	43	6	2	1
Losartan	0	0	71	21	13	13
Metformin	21	17	425	106	88	67
Metoprolol	0	0	78	15	14	14
Omeprazole	5	0	288	63	59	54
Paracetamol	4	2	198	33	26	22
Ramipril	0	0	136	27	23	23
Salbutamol	1	0	135	14	12	11
Simvastatin	73	73	120	152	93	20
Total	184	171	3,143	763	573	389
Median	1	0	125	24	20	19

Supplementary Table 6. Significant drug and multi-omics associations. Number of significant drug multi-omics associations identified when applying T-test and ANOVA to the residualized data compared to using the MOVE framework with T-test or Bayes test. Significance was set to estimated ground truth FDR 0.05 and statistical tests used were two-sided.

Drug	Cluster A n=103	Cluster B n=22	Cluster C n=84	Cluster D n=45	Mixed cluster n=472	Total signif omics	% with archetype
Acetylsalicylic Acid	3	0	1	0	0	39	7.7
Amlodipine	7	0	1	1	0	48	16.7
Atenolol	0	0	0	0	0	13	0.0
Atorvastatin	1	0	0	1	0	45	4.4
Bendroflumethiazide	0	1	1	0	0	13	15.4
Bisoprolol	1	0	0	0	0	31	3.2
Codeine	1	0	0	1	0	7	28.6
Enalapril	0	0	0	0	0	1	0.0
Hydrochlorothiazide	0	0	0	0	0	19	0.0
Lansoprazole	0	0	0	0	0	7	0.0
Levothyroxine Sodium	1	0	0	0	0	20	5.0
Lisinopril	0	0	0	0	0	2	0.0
Losartan	1	1	0	0	0	13	7.7
Metformin	6	1	2	0	0	88	9.1
Metoprolol	0	0	0	0	0	14	0.0
Omeprazole	4	0	3	1	0	59	11.9
Paracetamol	1	0	1	1	0	26	7.7
Ramipril	0	1	0	1	0	23	8.7
Salbutamol	1	0	0	0	0	12	8.3
Simvastatin	2	0	4	0	0	93	5.4

Supplementary Table 7. Stratification by archetype analysis from Wesolowska-Andersen and Brorsson et al. We investigated whether the significant drug-omics associations could be stratified by T2D subtype as inferred by archetype (cluster) analysis performed in Wesolowska-Andersen and Brorsson et al. The four archetypes A-D were defined as, Archetype A (n = 103): low BMI, older age, high insulin sensitivity and high cholesterol; Archetype B (n = 22): obese, insulin sensitive, favorable lipid profiles and low fasting creatinine levels; Archetype C (n = 84) obese, insulin resistance, dyslipidemia; Archetype D (n = 45) obesity, insulin resistance, dyslipidemia, low glucose control, low glucose sensitivity; Mix (n = 472): individuals not associated with any of the archetypes (in between). Between 0-28.6% of the significant hits could be associated to a specific subtype, although the 28.6% were from codeine with only 7 total hits. A median of 6.5% of the hits could be assigned to a specific disease subtype.

**Curated drug name**	**N total**	**N missing start date**	**Median years**	**Quantile 2.5**	**Quantile 97.5**
Acetylsalicylic Acid	129	3	5.9	0.5	22
Amlodipine	105	6	3.2	0.2	25.7
Atenolol	59	1	11.5	1.2	28.6
Atorvastatin	146	2	1.7	0.1	18.2
Bendroflumethiazide	82	6	5.2	0.5	25.2
Bisoprolol	82	1	3	0.2	15.9
Codeine	46	4	3.2	0.2	22.5
Enalapril	47	0	4.8	0.7	21.2
Hydrochlorothiazide	87	0	5	0.7	22.8
Lansoprazole	57	4	4.1	0.1	16.6
Levothyroxine Sodium	76	2	8.2	0.8	34.4
Lisinopril	46	4	3.2	0.2	22.5
Losartan	58	6	3.8	0.1	16.9
Metformin	277	6	1.1	0.2	2.1
Metoprolol	57	4	4.9	1	19.9
Omeprazole	125	5	5.1	0.3	20.9
Paracetamol	90	9	3.4	0.2	24.1
Ramipril	96	0	3.2	0.3	16.7
Salbutamol	45	2	5.2	0.4	46.3
Simvastatin	323	9	1.9	0.1	13.6

Supplementary Table 8. Overview of medication usage for the cohort. Number of individuals taking a drug and the length distribution in years that they have been administered the drug in years (2.5% quantile, median, 97.5% quantile) when the measurements (cohort baseline) were taken. The individuals with 'N missing start date' where not included in the calculation of the length of duration.

Dataset 1	Dataset 2	Mean Difference	P-adj	Lower	Upper
Diet and wearables	Clinical continuous	-0.00136878	1.98E-05	-0.0021664	-0.0005711
Metagenomics	Clinical continuous	-0.00402892	0	-0.0048266	-0.0032313
Metagenomics	Diet and wearables	-0.00266015	0	-0.0034578	-0.0018625
Metagenomics	Proteomics	-0.00201559	1.20E-10	-0.0028133	-0.0012179
Metagenomics	Targeted metabolomics	-0.00247546	0	-0.0032731	-0.0016778
Metagenomics	Transcriptomics	1.14E-05	1	-0.0007862	0.00080911
Metagenomics	Untargeted metabolomics	-0.003326	0	-0.0041237	-0.0025283
Proteomics	Clinical continuous	-0.00201333	1.25E-10	-0.002811	-0.0012157
Proteomics	Diet and wearables	-0.00064455	0.19884057	-0.0014422	0.00015312
Targeted metabolomics	Clinical continuous	-0.00155346	8.24E-07	-0.0023511	-0.0007558
Targeted metabolomics	Diet and wearables	-0.00018468	0.99278834	-0.0009824	0.00061299
Targeted metabolomics	Proteomics	0.000459869	0.59991517	-0.0003378	0.00125754
Transcriptomics	Clinical continuous	-0.00404037	0	-0.004838	-0.0032427
Transcriptomics	Diet and wearables	-0.00267159	0	-0.0034693	-0.0018739
Transcriptomics	Proteomics	-0.00202704	9.49E-11	-0.0028247	-0.0012294
Transcriptomics	Targeted metabolomics	-0.00248691	0	-0.0032846	-0.0016892
Transcriptomics	Untargeted metabolomics	-0.00333744	0	-0.0041351	-0.0025398
Untargeted metabolomics	Clinical continuous	-0.00070293	0.12297685	-0.0015006	9.47E-05
Untargeted metabolomics	Diet and wearables	0.000665852	0.16801066	-0.0001318	0.00146352
Untargeted metabolomics	Proteomics	0.001310403	5.13E-05	0.00051273	0.00210807
Untargeted metabolomics	Targeted metabolomics	0.000850534	0.02850969	5.29E-05	0.0016482

Supplementary Table 9. Average drug effect sizes between omics datasets. We used two-sided ANOVA and Tukey Honest Significant Difference (HSD) tests to identify significant differences of the average drug effects between the omics datasets. P-adj indicates p-value after adjustment for multiple comparisons at 0.95 confidence level. Lower and Upper indicates the lower and upper end points of the intervals in the difference between the means of Dataset 1 and Dataset 2.

**Dataset**	**PCC (difference)**	**PCC (cosine)**
Clinical continuous	-0.14	0.02
Diet and wearables	0.18	0.01
Proteomics	-0.02	0.01
Targeted metabolomics	-0.15	0.00
Untargeted metabolomics	0.09	0.03
Transcriptomics	0.16	-0.04
Metagenomics	0.00	0.00

Supplementary Table 10. Uncertainty of modalities does not influence inferred effect sizes. The difference between observations (residualized data input) and reconstructions (output from the VAE) can be seen as a measure of uncertainty in the modalities, and we therefore investigated if this would influence the effect sizes inferred by MOVE. We therefore compared, using Pearson Correlation Coefficient (PCC), the absolute differences between observations and reconstructions (“difference”) to the inferred effect sizes of the drugs. Similarly, we compared the cosine distances between input and reconstructions (“cosine”, similar to Supplementary Figure 5) and the inferred effect sizes of the drugs. Only small correlations were found indicating that uncertainties in the modalities are not driving the inferred effect sizes.

Source	No. of drugs	No. of DDIs	Link
Drugbank	3,779	2,255,390	https://go.drugbank.com/releases/latest
Twosides	1,951	351,647	https://tatonettilab.org/offsides/
TRANSFORMER	1,050	323,911	https://bioinformatics.charite.de/transformer
KEGG	2,963	206,875	https://www.genome.jp/kegg/drug/
Interaksjoner	3,364	194,933	https://legemiddelverket.no/andre-temaer/fest
SemMedDB	2,455	83,458	https://lhncbc.nlm.nih.gov/ii/tools/SemRep_SemMedDB_SKR.html
HIV	1,339	34,634	https://www.hiv-druginteractions.org/
CANCER	1,021	30,256	https://cancer-druginteractions.org/
ONC NI	509	21,705	https://github.com/dbmi-pitt/public-PDDI-analysis
Interaktion Databasen	932	18,691	https://laegemiddelstyrelsen.dk/da/bivirkninger/interaktionsdatabasen-og-medicinkombination/
Flockchart	289	18,400	https://drug-interactions.medicine.iu.edu/MainTable.aspx
HEP	1,339	17,970	https://www.hep-druginteractions.org/
NDF-RT	1,144	12,199	https://evs.nci.nih.gov/ftp1/NDF-RT/
DDI Corpus 2013	1,307	10,857	https://github.com/isegura/DDICorpus
DDI Corpus 2011	846	4,664	https://github.com/isegura/DDICorpus
OSCAR	158	3,378	https://sourceforge.net/projects/oscarmcmaster/
CIMA	448	3,250	https://cima.aemps.es/cima/publico/nomenclator.html
PK Corpus	451	3,211	https://github.com/dbmi-pitt/public-PDDI-analysis
ONC HIGH	195	2,865	https://github.com/dbmi-pitt/public-PDDI-analysis
NLM CV Corpus	458	2,861	https://github.com/dbmi-pitt/public-PDDI-analysis
UHN	103	1,855	https://hivclinic.ca/drug-information/drug-interaction-tables/
Crediblemeds	163	403	https://www.crediblemeds.org/index.php/healthcare-providers/common-drug-interactions
PHAEDRA Corpus	126	219	http://www.nactem.ac.uk/PHAEDRA/index.php
HIV-insite	108	209	https://hivinsite.ucsf.edu/
DDINCBI	177	201	https://sourceforge.net/projects/bioc/files/

Supplementary Table 11. Overview of drug-drug interaction databases used. DDI: Drug-drug Interaction.
