# Supplementary material for: Discovery of drug–omics associations in type 2 diabetes with generative deep-learning models
Source: Nat Biotechnol. 2023 Jan 2;41(3):399–408. doi: 10.1038/s41587-022-01520-x (PMC10017515; doi:10.1038/s41587-022-01520-x)
Supplement: Supplementary file 2 — Reporting Summary [file 41587_2022_1520_MOESM2_ESM.pdf]

nature research

Corresponding author(s): Simon Rasmussen & Søren Brunak

Last updated by author(s): Sep 16, 2022

Reporting Summary

Nature Research wishes to improve the reproducibility of the work that we publish. This form provides structure for consistency and transparency in reporting. For further information on Nature Research policies, see our Editorial Policies and the Editorial Policy Checklist.

Statistics

For all statistical analyses, confirm that the following items are present in the figure legend, table legend, main text, or Methods section.

n/a	Confirmed	
☐	☒	The exact sample size (*n*) for each experimental group/condition, given as a discrete number and unit of measurement
☐	☒	A statement on whether measurements were taken from distinct samples or whether the same sample was measured repeatedly
☐	☒	The statistical test(s) used AND whether they are one- or two-sided<br>*Only common tests should be described solely by name; describe more complex techniques in the Methods section.*
☐	☒	A description of all covariates tested
☐	☒	A description of any assumptions or corrections, such as tests of normality and adjustment for multiple comparisons
☐	☒	A full description of the statistical parameters including central tendency (e.g. means) or other basic estimates (e.g. regression coefficient) AND variation (e.g. standard deviation) or associated estimates of uncertainty (e.g. confidence intervals)
☐	☒	For null hypothesis testing, the test statistic (e.g. *F*, *t*, *r*) with confidence intervals, effect sizes, degrees of freedom and *P* value noted<br>*Give P values as exact values whenever suitable.*
☐	☒	For Bayesian analysis, information on the choice of priors and Markov chain Monte Carlo settings
☒	☐	For hierarchical and complex designs, identification of the appropriate level for tests and full reporting of outcomes
☐	☒	Estimates of effect sizes (e.g. Cohen's *d*, Pearson's *r*), indicating how they were calculated

Our web collection on statistics for biologists contains articles on many of the points above.

Software and code

Policy information about availability of computer code

Data collection	No software were used for data collection in this study. Please refer to data section for more detail.
Data analysis	RailRNA v.0.2.4b<br>PyTorch v.1.7.0<br>CUDA v.10.2.89<br>SciPy v.1.3.1<br>Reactome v3.7<br>WebGestaltR v.0.4.4<br>MetaboAnalyst v.5<br>MOVE: https://github.com/RasmussenLab/MOVE

For manuscripts utilizing custom algorithms or software that are central to the research but not yet described in published literature, software must be made available to editors and reviewers. We strongly encourage code deposition in a community repository (e.g. GitHub). See the Nature Research guidelines for submitting code & software for further information.

Data

Policy information about availability of data

All manuscripts must include a data availability statement. This statement should provide the following information, where applicable:

- Accession codes, unique identifiers, or web links for publicly available datasets
- A list of figures that have associated raw data
- A description of any restrictions on data availability

Due to the informed consent given by study participants, the various national ethical approvals for the present study, and the European General Data Protection

Regulation (GDPR), individual-level clinical and omics data cannot be transferred from the centralized IMI-DIRECT repository. Requests for access to summary statistics IMI-DIRECT data, including those presented here, can be made to DIRECTdataaccess@Dundee.ac.uk. Requesters will be informed on how summary-level data can be accessed via the DIRECT secure analysis platform following submission of appropriate application. The IMI-DIRECT data access policy is available at https://directdiabetes.org. Example data is available at https://github.com/RasmussenLab/MOVE/ for testing of MOVE. As described in the methods section we used Anatomical Therapeutic Chemical Classification (ATC, https://www.who.int/tools/atc-ddd-toolkit/atc-classification), WebGestalt (v.0.4.4 at http://www.webgestalt.org for analysis of gene ontologies, Reactome (v.3.7 at https://reactome.org) for analysis of molecular pathways, and MetaboAnalyst (v.5 at https://www.metaboanalyst.ca) for analysis of targeted metabolomics data. The 25 databases of drug-drug interactions (DDI) are listed in Supplementary Table 11.

Field-specific reporting

Please select the one below that is the best fit for your research. If you are not sure, read the appropriate sections before making your selection.

☒ Life sciences ☐ Behavioural & social sciences ☐ Ecological, evolutionary & environmental sciences

For a reference copy of the document with all sections, see nature.com/documents/nr-reporting-summary-flat.pdf

Life sciences study design

All studies must disclose on these points even when the disclosure is negative.

Sample size	A total of 789 individuals with newly diagnosed type 2 diabetes were recruited at six centers in Europe (Amsterdam, Copenhagen, Dundee, Exeter, Lund and Newcastle). This was all the individuals with newly diagnosed T2D that were included in the cohort.
Data exclusions	For transcriptomics data only genes with a standard deviation higher than average standard deviation of all genes were included.
Replication	The findings made from the DIRECT newly diagnosed type 2 diabetes cohort were not replicated in another cohort as no cohort with similar data exist.
Randomization	All participants had newly diagnosed type 2 diabetes and were therefore not randomized. In the proteomics data, all measurements within the measurable range based on the OLINK antibody panel were included and residualized for plate layout. The continuous data were residualized by the collection center as the data was collected from six different European countries and, thus, handled by different nurses and lab technicians, as well as differences in the time-of-day samples were taken, which could have a large effect on the measurements. Additionally, the data were residualized for age and sex as these could be biological non-disease-related confounders in the data. Lastly, each continuous data set was Z-scale normalized per feature to ensure that each feature was distributed around zero.
Blinding	The investigators were not blinded as all individuals were newly diagnosed type 2 diabetes patients.

Reporting for specific materials, systems and methods

We require information from authors about some types of materials, experimental systems and methods used in many studies. Here, indicate whether each material, system or method listed is relevant to your study. If you are not sure if a list item applies to your research, read the appropriate section before selecting a response.

Materials & experimental systems

n/a	Involved in the study	
☒	☐	Antibodies
☒	☐	Eukaryotic cell lines
☒	☐	Palaeontology and archaeology
☒	☐	Animals and other organisms
☐	☒	Human research participants
☒	☐	Clinical data
☒	☐	Dual use research of concern

Methods

n/a	Involved in the study	
☒	☐	ChIP-seq
☒	☐	Flow cytometry
☒	☐	MRI-based neuroimaging

Human research participants

Policy information about studies involving human research participants

Population characteristics	The participants were recruited from six different locations in Northern Europe, The Netherlands, UK, Denmark and Sweden. Full information can be found in Koivula et al., Diabetologia. 2019. https://dx.doi.org/10.1007%2Fs00125-019-4906-1
Recruitment	In brief, we used the newly-diagnosed sub-cohort of the IMI-DIRECT study consisting of 789 participants, with mean age at inclusion of 62 years and the youngest at 35 years. Participants were diagnosed within two years before recruitment and had glycated hemoglobin (HbA1c) < 60.0 mmol/mol (< 7.6%) within the previous three months. Full information can be found in Koivula et al., Diabetologia. 2019. https://dx.doi.org/10.1007%2Fs00125-019-4906-1
Ethics oversight	Approval for the study protocol was obtained from each of the regional research ethics review boards separately (Lund,

Ethics oversight	Sweden: 20130312105459927, Copenhagen, Denmark: H-1-2012-166 and H-1-2012-100, Amsterdam, Netherlands: NL40099.029.12, Newcastle, Dundee and Exeter, UK: 12/NE/0132) and all participants provided written informed consent at enrolment.

Note that full information on the approval of the study protocol must also be provided in the manuscript.
